# A smartphone readout system for gold nanoparticle-based lateral flow assays: application to monitoring of digoxigenin

**DOI:** 10.1007/s00604-018-3195-6

**Published:** 2019-01-19

**Authors:** Christoph Ruppert, Navneet Phogat, Stefan Laufer, Matthias Kohl, Hans-Peter Deigner

**Affiliations:** 1 Medical and Life Sciences Faculty, Furtwangen University, Jakob-Kienzle Str. 17, 78054 Villingen-Schwenningen, Germany; 2 Institute of Precision Medicine, Furtwangen University, Jakob-Kienzle Str. 17, 78054 Villingen-Schwenningen, Germany; 3 Department of Pharmaceutical Chemistry, Pharmaceutical Institute, University of Tuebingen, Auf der Morgenstelle 8, 72076 Tuebingen, Germany; 4 EXIM Department, Fraunhofer Institute IZI, 10057 Rostock, Germany

**Keywords:** Lateral flow assay, Point-of-care diagnostics, Smartphone imaging, Nanoparticles, Image processing, R-package, Shiny app

## Abstract

For modern approaches in precision medicine, fast and easy-to-use point-of-care diagnostics (POCs) are essential. Digoxin was chosen as an example of a drug requiring close monitoring. Digoxin is a cardiac glycoside used for the treatment of tachycardia with a narrow therapeutic window of 0.5–2.0 ng·mL^−1^, and toxic effects are common for concentrations above 2.5 ng·mL^−1^. For monitoring of blood concentration levels and treatment of intoxication, highly selective antibodies for digoxin and its hapten, digoxigenin, are available. A smartphone readout system is described for measuring digoxigenin in human serum using a common gold nanoparticle lateral flow assay (LFA). The R-package GNSplex, which also includes a Shiny app for quantitative test interpretation based on linear models, is used for image analysis. Images of lateral flow strips were taken with an iPhone camera and a simple darkbox made from black cardboard. Sensitivity and accuracy of the quantitative smartphone system as well as analytical parameters such as limit of detection (LOD) were determined and compared to data obtained with a high resolution BioImager. The data show that the smartphone based digoxin assay yields reliable quantitative results within the clinically relevant concentration range.

**Electronic supplementary material:**

The online version of this article (10.1007/s00604-018-3195-6) contains supplementary material, which is available to authorized users.

**Graphical abstract:**
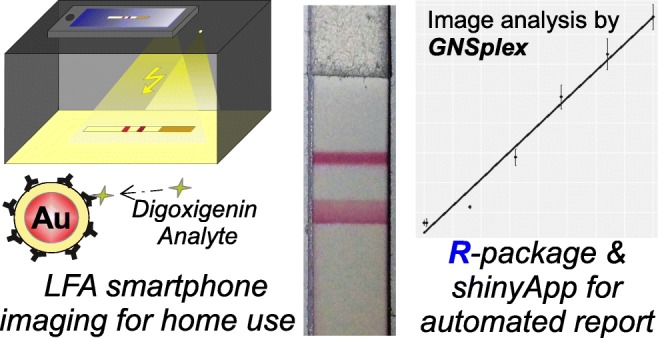
For therapeutic drug monitoring and point of care diagnostics we introduce the open source R-package GNSplex for smartphone readout and interpretation of lateral flow assays. The cardiac glycoside dixogin was used as target for this quantitative smartphone reader

## Introduction

An important aspect of precision medicine is the measurement of drug, metabolite and biomarker concentrations at high frequencies, e.g., from blood or plasma, for diagnosis and control of therapeutic dosages. This measurement can be critical for matching the therapeutic window concentrations and for decreasing physical stress for the treated patient, especially for potentially toxic drugs with severe adverse effects. For example, organ transplant patients with immunosuppressive medications or HIV-infected people on anti-retroviral treatments are groups that benefit from high frequency therapeutic drug monitoring (TDM) [1, 2]. Fast and easy-to-use point-of-care devices and diagnostics (POCs) that can be performed by the patient are required to address this need. These self-monitoring tests must be cost-effective and would preferably not require additional hardware for measurement and data processing.

Here, a gold nanoparticle lateral flow assay (LFA) for digoxigenin was used to establish a smartphone-based readout system for home monitoring of cardiac glycoside digoxin. Digoxin is a cardiac glycoside used for treatment of tachycardia with a narrow therapeutic window of 0.5–2.0 ng·mL^−1^; at serum concentrations above 2.5 ng·mL^−1^, intoxication is likely. Digoxin is frequently administered in combination with beta-blockers, and control of blood concentrations is important [3, 4]. Monitoring of digoxin levels in patients is common practice and is usually performed through quantitative ELISA.

**Fig. 1:**
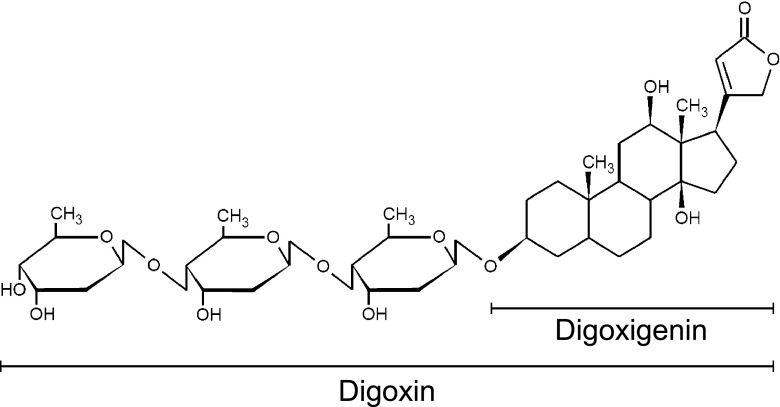
Structure of target drug digoxin and its derivative digoxigenin (hapten for antibody coupling)

The binding epitope of most highly selective antibodies used in digoxin assays is digoxigenin, which is a deglycosylated derivative (Fig. 1). Digoxigenin is widely used as a hapten or binding tag for immunoassays, allowing transfer of our system to further diagnostic targets.

**Fig. 2:**
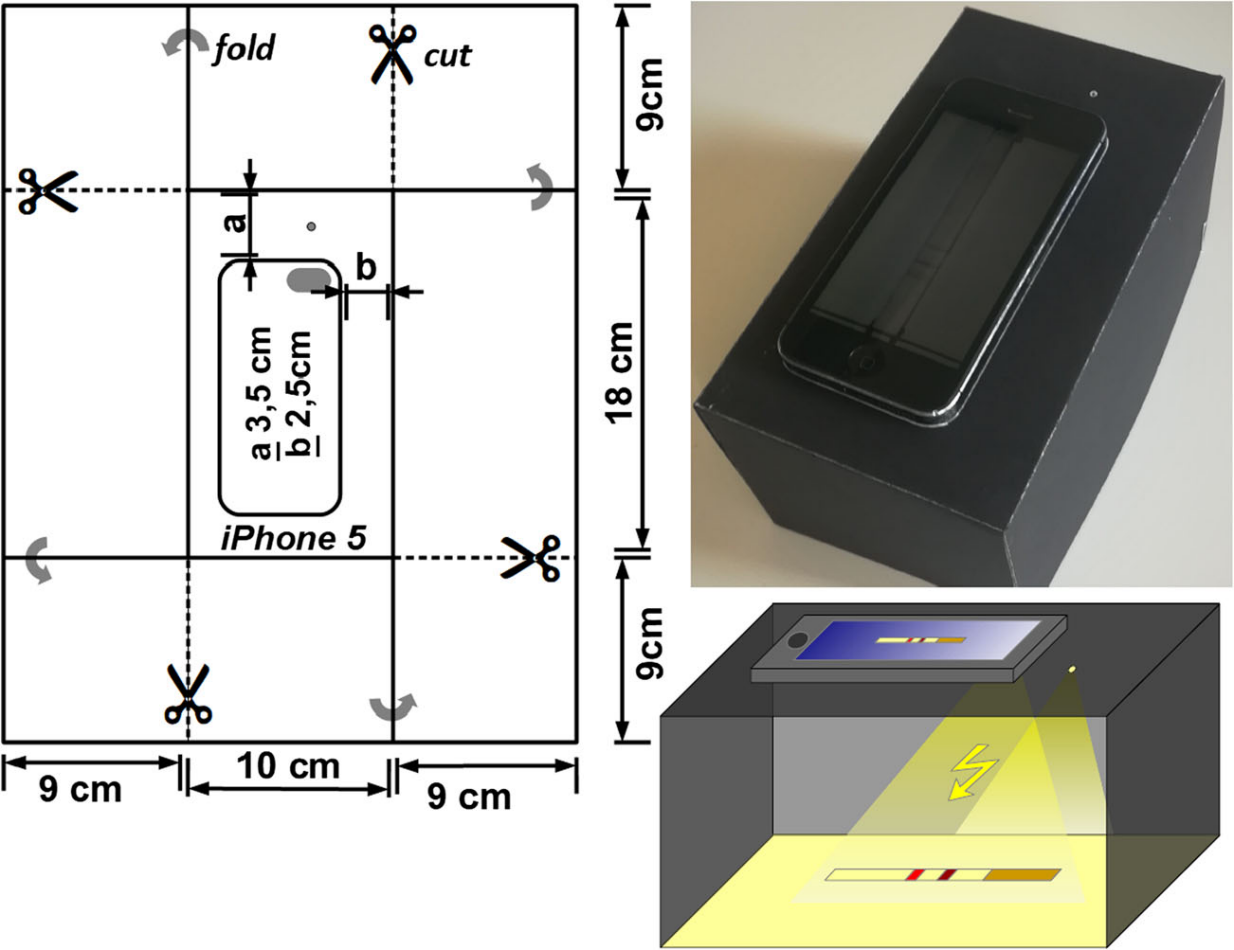
Cutting pattern for the darkbox from black cardboard with dimensions; picture of darkbox for smartphone (iPhone 5 s) imaging; illustration of darkbox for smartphone imaging. Pictures were taken in standard settings with a flashlight. For adjustment of the built-in autofocus, a 1 mm hole allowing little external light is also included in the topside of the darkbox

Lateral flow immunoassays are currently used for qualitative, semiquantitative and, to some extent, quantitative applications, including a widespread distribution in nonlab environments [5]. Imaging and readout hardware is available but far from being affordable for home use. The first smartphone approaches for point-of-care diagnostics usually involved additional attachment parts or special posttreatment of the used LFAs. Zangheri et al. described a chemiluminescence-based lateral flow method for cortisol sensing in saliva. The method employs a 3D-printed ABS plastic accessory with an incorporated lens and can reach detection limits as low as 0.3 ng·mL^−1^ [6]. Mudanyali and colleagues reported on a smartphone-based platform with a 3D-printed accessory including incorporated LEDs for illumination to obtain qualitative lateral flow assay results [7]. Several more approaches, such as a study by You et al., use smartphone readers for detection of thyroid-stimulating hormone, and work by Lee et al. used this method for detection of Aflatoxin B1. However, these approaches all use customized add-ons [8, 9]. For readout of our lateral flow assays, we use an iPhone 5S in combination with a simple darkbox made from black cardboard (Fig. 2).

To increase the scope and statistical power of the collected quantitative data, we developed an algorithm in the statistical software R including the colorimetric readout of test strips and tools for background- and baseline correction. We found that this setup affords viable quantitative data, similar to results acquired by a high performance BioImager System as a reference. To further increase the usability of our approach, we generated the R-package GNSplex including the algorithm for data processing; to simplify access to our algorithm, we added a Shiny app to ouR-package.

## Experimental

### Reagents and materials

Digoxigenin, BSA (bovine serum albumin), human serum, Tween-20, EDC **(***N*-(3-Dimethylaminopropyl)-*N*′-ethylcarbodiimide hydrochloride)**,** Sulfo-NHS (sulfosuccinimidyl 4,4′-azipentanoate), BIS-TRIS hydrochloride (2,2-Bis(hydroxymethyl)-2,2′,2″-nitrilotriethanol), sodium chloride, Triton X-100, HEPES (4-(2-hydroxyethyl)-1-piperazineethanesulfonic acid) and Pur-A-Lyzer-Midi (6000-8000MWCO) were purchased from Sigma Aldrich (https://www.sigmaaldrich.com). PBS (Phosphate buffered saline 10×) was purchased from AppliChem (https://www.itwreagents.com). α-digoxigenin-monoclonal-antibodies were purchased from Roche (https://lifescience.roche.com). Spherical gold nanoparticles, 50 nm Lipoic Acid NanoXact Gold, were purchased from nanoComposix (https://nanocomposix.eu/). Lateral-Flow-Assay strips for the LFA were provided by R-Biopharm (https://r-biopharm.com/de). The test line (tl) consists of a digoxigenin-BSA-conjugate. The control line (cl) consists of anti-mouse-polyclonal antibody. Test and control lines are 5 mm apart. The membrane material is nitrocellulose with 0.5 μm pore size. Buffers and reagents were prepared in milli-Q water (18.2 MΩ·cm).

### Immunoprobe composition and synthesis

#### Synthesis of spherical gold bioprobes

A 1 μL quantity of a freshly prepared EDC/sulfo-NHS solution (c = 5.5 mmol·L^−1^ in milliQ water, 100.000-fold molar excess) was added to 1 ml of 50 nm Lipoic Acid NanoXact Gold particles (c = 56.5 pmol) and incubated at 250 rpm, 7 °C for 30 min in the dark. A dialysis tube (MWCO 6000-8000 kDa) was used to remove excess of EDC/sulfo-NHS. The mixture was dialyzed against 800 ml HEPES-buffer (20 mM 0.1% Tween 20 pH 7.2) for 90 min, at room temperature (RT), in the dark. Then, a 120-fold excess of Digoxigenin-antibody (1 μL c = 6.6 μmol·L^−1^) was added. The reaction mix was incubated for 2 h, 250 rpm, 7 °C in the dark. Then, 10% BSA-solution in HEPES was added to a final content of 1%. The volume of AuNP-conjugate solution was adjusted to a final volume of 1.5 mL with 1% BSA in HEPES. Conjugates obtained can be stored at 4 °C in the dark for several weeks.

### LFA assay procedure, samples and reagents

Digoxigenin stock solutions for calibration with concentrations of 0,1,20,40,60,80,100 nmol·L^−1^ were prepared in PBS (pH 7.4, 0.01 M; 0.001 M EDTA). Every calibration solution was measured in five repetitions. Digoxigenin samples in human serum were prepared at concentrations of 0, 1, 5, 10, 15, 20, 25, 30, 40, 60, 80, 100 nmol·L^−1^ (three repetitions). The running buffer (RB) contained 0.05 M BIS-TRIS, 8% Triton X-100, 0.3% BSA.

A 10 μL sample (digoxigenin calibration solution or serum sample), 45 μL of running buffer, and 15 μL of conjugate solution were mixed in a 2 mL flat bottom reaction vessel; 30 s after mixing, the components the Digoxigenin LFA-strip were placed in the sample mix. The runtime for the 70 μL sample mix was 5 min. The test-strips were then placed on a flat surface and allowed to dry for 10 min. LFA-strips then were then ready for readout through the iPhone 5S or BioImager (ChemStudio Plus, Analytic Jena).

### LFA assay principle and format

A direct competitive digoxigenin-LFA assay based on colorimetric bioprobes (gold nanoparticle-antibody-conjugates) was used in the detection method. Digoxigenin calibration solutions or spiked samples in human serum and antibody conjugates were mixed and applied on the LFA-membranes. Bioprobes without bound digoxigenin were bound to the test line. Otherwise, if digoxigenin was bound to the conjugated antibodies on the nanoparticle surface, the probes migrated further to the control line, where anti-mouse secondary antibodies bound to the mouse primary antibodies from the bioprobes. The colored bands on the test/control lines are photographed (in our case, using a CMOS smartphone camera from the iPhone 5S and a cardboard darkbox or BioImager (ChemStudio Plus with a 16MP CCD camera).

**Fig. 3:**
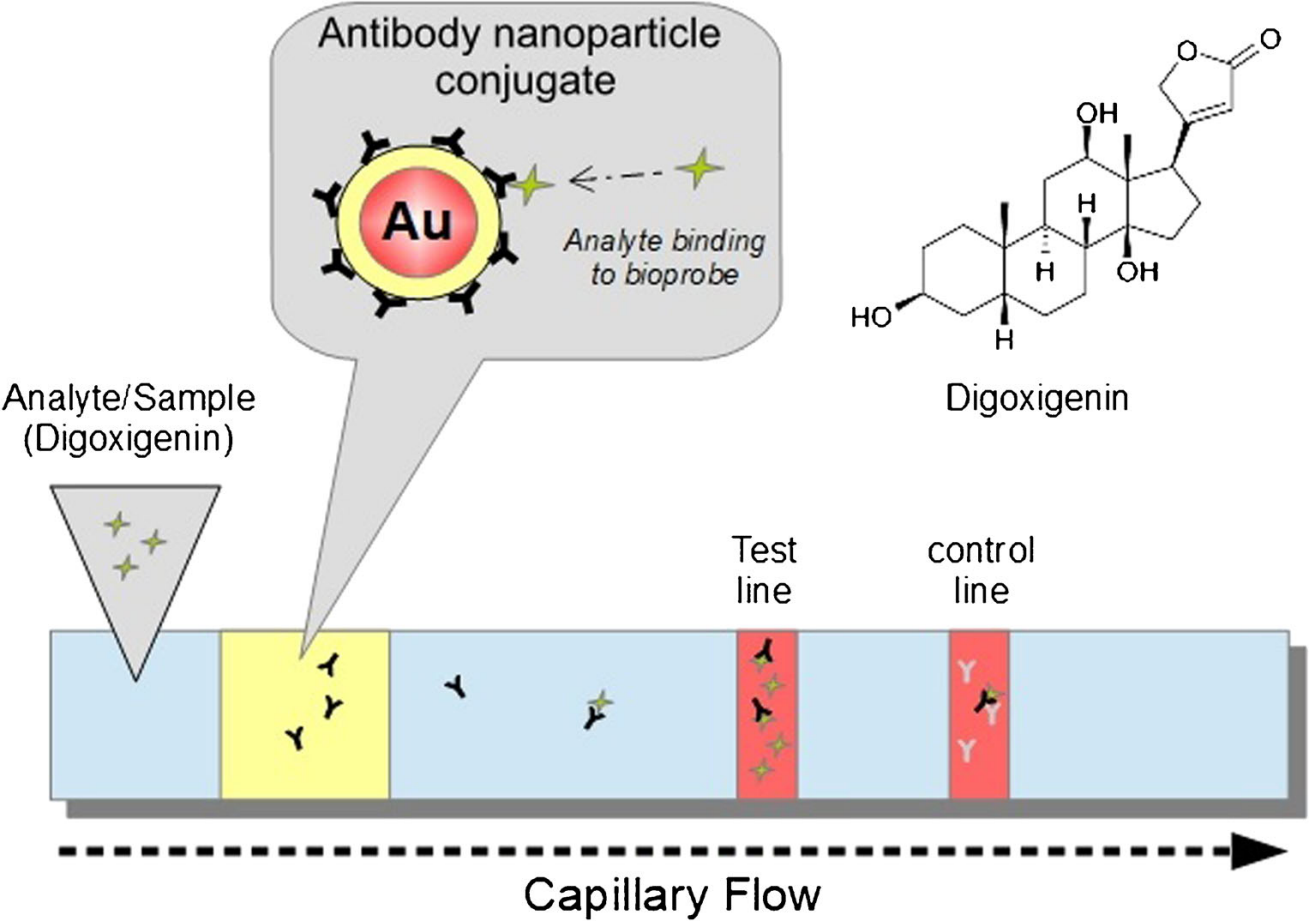
Illustration of competitive lateral flow immunoassay for detection of digoxigenin. Test line/control line consist of digoxigenin-BSA-conjugate/anti mouse secondary antibodies. Sample consist of digoxigenin sample/running buffer/AuNP-anti digoxigenin-Ab–conjugates

The colorimetric signal on the test-line is inversely related to the digoxigenin concentration in the samples. With the control-line as a calibration standard, signals can be normalized and calibrated for different readout devices using the same statistical methods (see: Data acquisition and processing) (Fig. 3).

### GNSplex: an R-package for analysis of the data of gold nanoparticle-based bioassay

*GNSplex* is an open-source package completely developed in the statistical software R [10]. It is mainly based on the bioconductor package *EBImage* as well as R-packages *ggplot2 and ggpmisc* [10–13]. *GNSplex* utilizes the implemented functionalities of *EBImage* to process jpeg images from lateral flow strips cut to a specific size. Images must include the test and control line to obtain an appropriate signal. We provide templates of the jpeg files in the folder *exData* of our package *GNSplex*. *GNSplex* uses the R-packages *ggplot2* and *ggpmisc* to generate plots of the fitted linear models [10, 13]. The sources of *GNSplex* are available for download from https://github.com/NPhogat/GNSplex and can be installed in R using the package *devtools* [14, 15]. The package also includes an in-built *Shiny app* and a standalone graphical user interface (GUI) to make the analysis of the image data more user-friendly. In addition, the *Shiny app* can be used to generate an analysis report of the results via the R-package *rmarkdown* [16, 17]. BioImager and iPhone images of samples at different concentrations of digoxigenin calibration standards and spiked human serum samples were taken. The intensities of the test line (tl) and control line (cl) were extracted, the background was corrected and the normalized intensities (cl/tl) were computed. Linear models based on the normalized intensities (cl/tl) and the concentrations (nM) were used. To increase the functionality of our package, it is possible to fit simple linear models based on the standardized intensities (tl/cl) and the concentrations (nM). The package furthermore includes functions also incorporated into the GUI to compute the standard deviation (SD) within replicates of the raw intensities of the control and test line, as well as of normalized and standardized intensities, confidence intervals of normalized and standardized intensities and the Pearson correlation of the normalized and standardized intensities with respect to their predicted values. Further, the package can be used to compute the limit of detection (LOD) and limit of quantification (LOQ) statistically, based on two different methods. The first method to compute the LOD and LOQ is based on the following formulas:

$$ {\displaystyle \begin{array}{l}\mathrm{LOD}=\mathrm{Mean}\ \mathrm{of}\ \mathrm{blank}\ \mathrm{data}+{3}^{\ast}\left(\mathrm{standard}\ \mathrm{deviation}\ \mathrm{of}\ \mathrm{blank}\ \mathrm{data}\right)\\ {}\mathrm{LOQ}=\mathrm{Mean}\ \mathrm{of}\ \mathrm{blank}\ \mathrm{data}+{10}^{\ast}\left(\mathrm{standard}\ \mathrm{deviation}\ \mathrm{of}\ \mathrm{blank}\ \mathrm{data}\right)\end{array}} $$

The formulas for the second method to compute the limit of blank (LOB), LOD and LOQ read:

$$ {\displaystyle \begin{array}{l}\mathrm{LOB}=\mathrm{Mean}\ \mathrm{of}\ \mathrm{the}\ \mathrm{blank}\ \mathrm{data}+{1.645}^{\ast}\left(\mathrm{standard}\ \mathrm{deviation}\ \mathrm{of}\ \mathrm{blank}\ \mathrm{data}\right)\\ {}\mathrm{LOD}=\mathrm{LOB}+{1.645}^{\ast}\left(\mathrm{standard}\ \mathrm{deviation}\ \mathrm{of}\ 1\mathrm{nM}\ \mathrm{sample}\ \mathrm{data}\right)\\ {}\mathrm{LOQ}=\mathrm{Mean}\ \mathrm{of}\ \mathrm{blank}\ \mathrm{data}+{10}^{\ast}\left(\mathrm{standard}\ \mathrm{deviation}\ \mathrm{of}\ \mathrm{blank}\ \mathrm{data}\right)\end{array}} $$

The respective results for standardized intensities are shown in the supplementary information.

## Results and discussion

### Data acquisition via smartphone and BioImager

For each analyte (digoxigenin) concentration, a data set of five test strips was prepared and pictures were taken either with a high class BioImager (ChemStudioPlus, 16MP CCD-camera, Analytik Jena) or with an iPhone 5S in standard settings, applying flashlight in the previously described cardboard-darkbox. The Test-line (tl) is comprised of digoxigenin-hapten, while the Control-line (cl) is comprised of the secondary antibody for mouse Fc-fragments. The assay format is competitive, so a high digoxigenin content in the samples corresponds to strongly colored (dark) test-lines and light-colored control lines (inverse test-line signal).

**Fig. 4:**
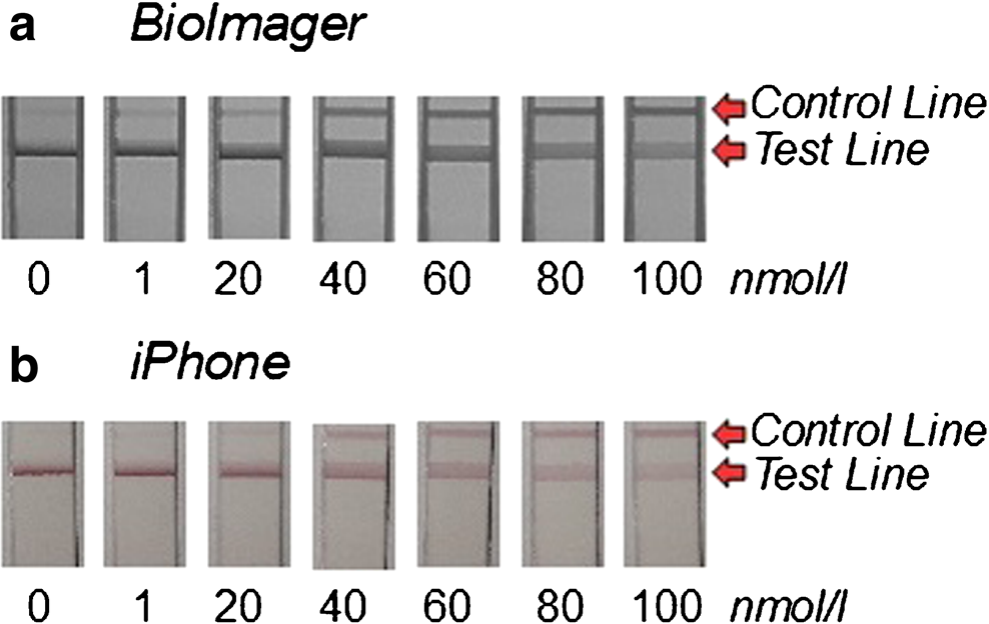
Data sets of pictures taken with **a** BioImager (ChemStudio PLUS, 16 MP CCD), **b** iPhone 5S (standard settings with flashlight); Data shows first set of Calibration standard tests-trips

As seen in the unprocessed pictures (Fig. 4), the high-resolution pictures produced by the BioImager are sharper and denser, which may be advantageous if a visual control by naked eye is intended. In particular, very low and very high concentrations are difficult to see in the smartphone pictures.

### Data processing

The main goal was to develop a user-friendly method, utilizing a smartphone, to compute digoxigenin concentrations from normalized intensities for potential use as a POC-device at home. Due to the narrow therapeutic range of digoxigenin, and based on the results of our experiments, simple linear models were used.

### Analysis of the lateral flow data of digoxigenin

**Fig. 5:**
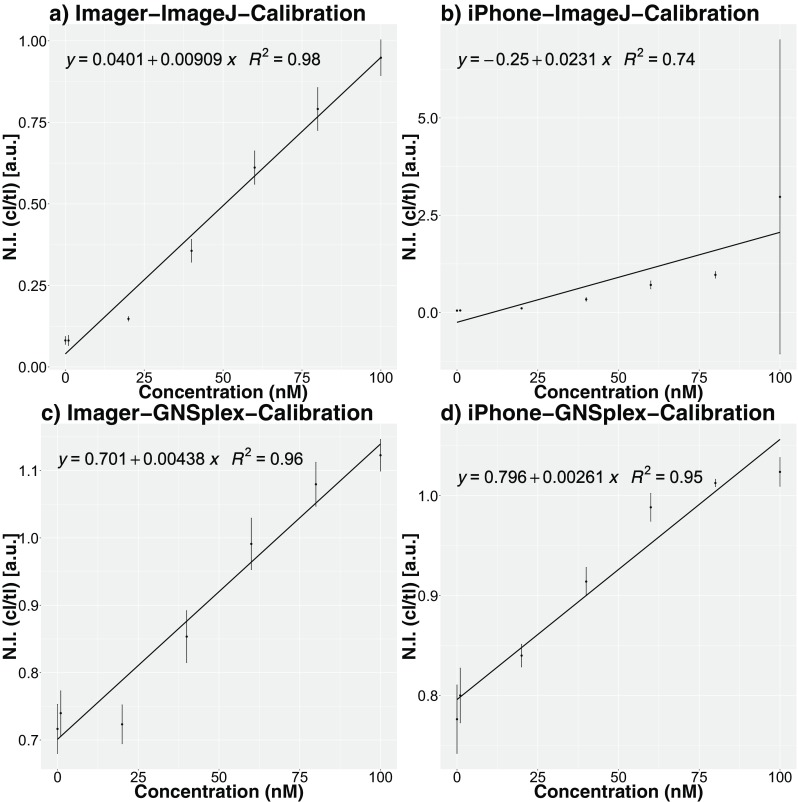
Concentration vs. normalized intensities (calibration standard digoxigenin) for readout trough ImageJ and GNSplex based on Imager and iPhone data, respectively; error bars represent the standard deviation of the normalized intensities within replicates

A common laboratory standard for colorimetric readout is the software *ImageJ*. We compare the normalized intensities and respective simple linear models computed for pure digoxigenin samples: one set of results was generated via *ImageJ* and a second set by the R-package *GNSplex*. The highest R^2^ of 0.98 (Fig. 5a) was obtained for the BioImager data processed through the *ImageJ* software, followed by an R^2^ of 0.96 (Fig. 5c) for the BioImager data processed with our R-package *GNSplex*. As depicted in Fig. 5b and d, the R-package, *GNSplex* works better for the iPhone data. The clearly weaker result for *ImageJ*, however, is mainly caused by the variable results measured for 100 nM. As expected, the results for the BioImager data are superior, while our R-package *GNSplex* works well; the results for the iPhone data are only slightly different from results for the BioImager data. Error bars in Fig. 5a, b, c and d represent the standard deviation of the normalized intensities within the replicates. The respective LOB (limit of blank), LOD (limit of detection) and LOQ (limit of quantification) calculated for the normalized intensities are given in Table 1. The standard deviation of the normalized intensities and the Pearson correlations between the normalized intensities and the predicted values are included in the supplementary information.

**Table 1:** LOD, LOQ and LOB for ImageJ and GNSplex readout based on Imager and iPhone data (calibration standard digoxigenin), respectively

ImageJ/GNSplex data processing normalized DIG calibration [nmol·L^−1^]	ImageJ 1st method		ImageJ 2nd method		GNSplex 1st method		GNSplex 2nd method	
	Imager	iPhone	Imager	iPhone	Imager	iPhone	Imager	iPhone
Limit of detection (LOD)	8.5	14.8	9.5	14.4	28.5	31.8	29.7	31.3
Limit of quantification (LOQ)	17.8	19.1	17.8	19.1	86.8	31.8	86.8	123.7
Limit of blank (LOB)	–	–	8.5	14.0	–	–	17.2	13.1

### Analysis of the lateral flow data of digoxigenin with serum sample

**Fig. 6:**
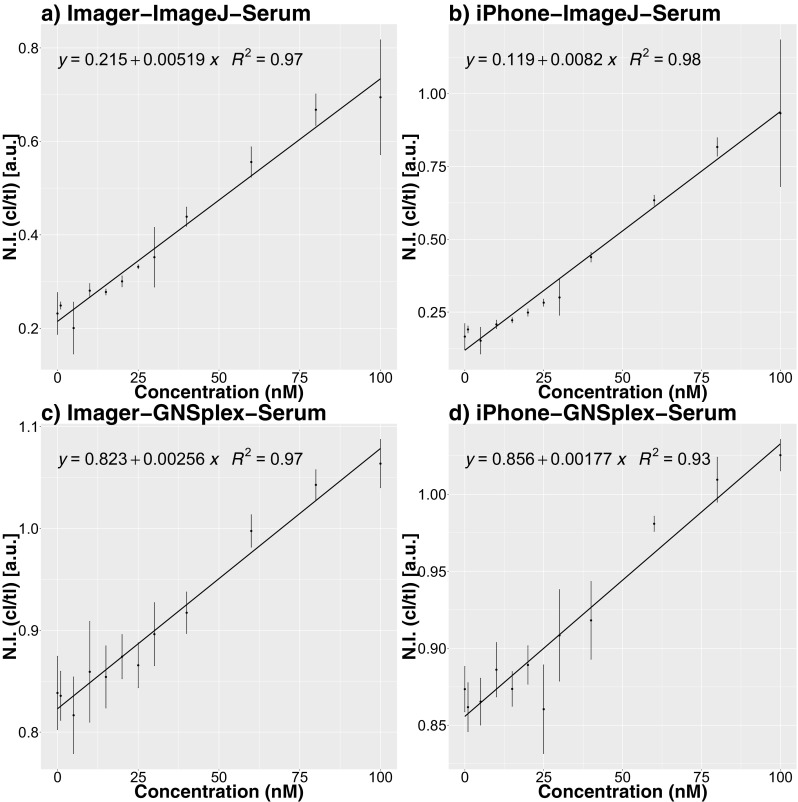
Concentration vs. normalized intensities (spiked serum digoxigenin) for readout through ImageJ and GNSplex with Imager or iPhone for readout based on Imager and iPhone data, respectively. The error bars given in the figures represent the standard deviation of the normalized intensities within replicates

**Fig. 7:**
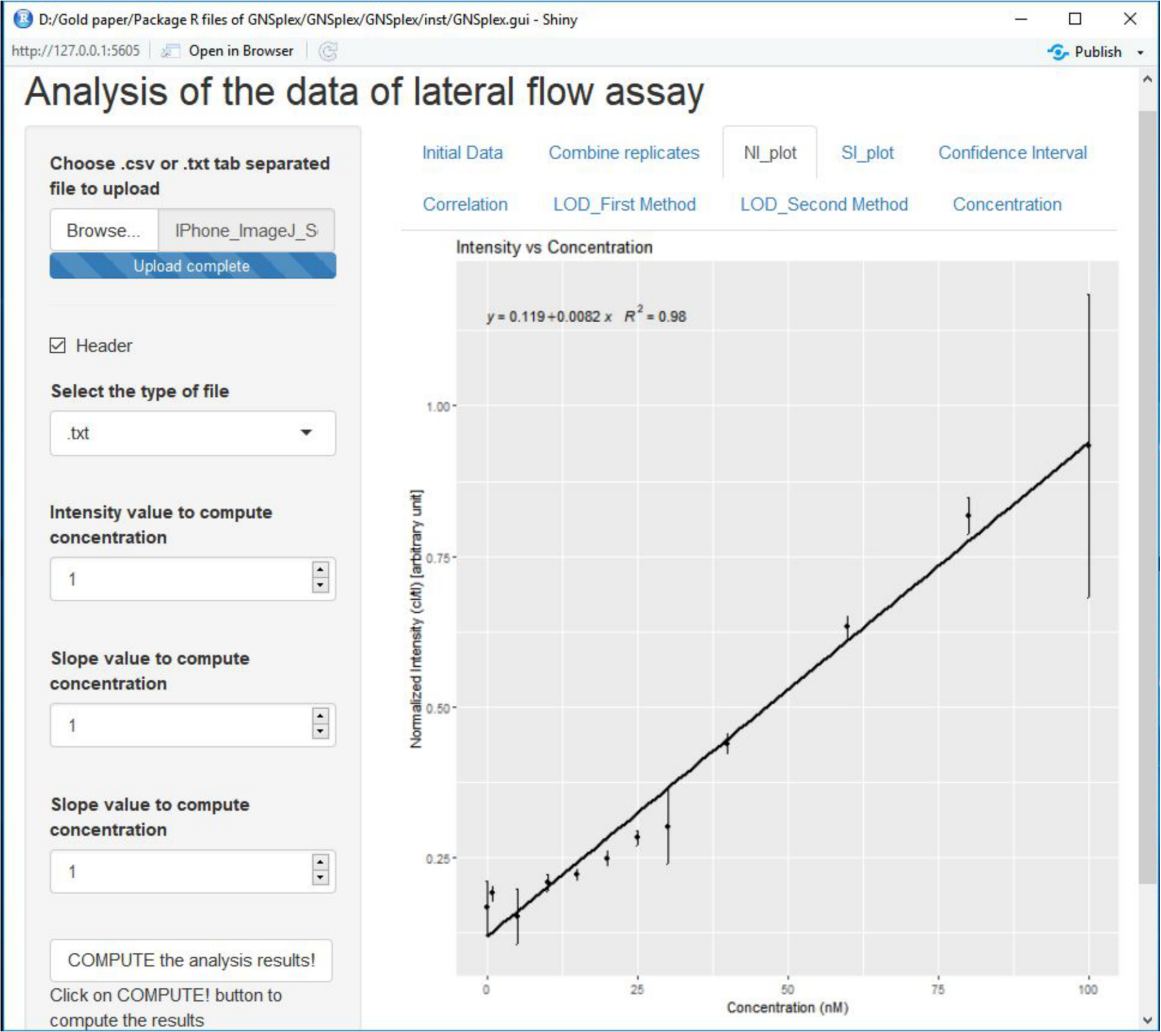
Screenshot of Shiny app for automatic data processing of lateral flow assays

This section compares the fitted simple linear regression models of the digoxigenin lateral flow assay with serum samples, where the images were processed via *ImageJ* and our R-package *GNSplex*. Figure 6 shows that the standard deviations within the replicates are smaller for ImageJ than those for *GNSplex*. However, there are high to very high R^2^ values in all cases. The results indicate that our smartphone-based system has the potential to achieve accuracies similar to those of a high-end imager system. The LOB, LOD and LOQ, computed by two different approaches, are shown in Table 2. The Pearson correlations between the normalized intensities and the predicted values are included into supplementary information (Fig. 7).

**Table 2:** LOD, LOQ and LOB for ImageJ and GNSplex readout based on Imager and iPhone data (spiked serum digoxigenin), respectively

ImageJ/GNSplex data processing normalized DIG Serum [nmol·L^−1^]	ImageJ 1st method		ImageJ 2nd method		GNSplex 1st method		GNSplex 2nd method	
	Imager	iPhone	Imager	iPhone	Imager	iPhone	Imager	iPhone
Limit of detection (LOD)	29.1	21.9	19.8	16.9	48.3	37.0	44.7	39.4
Limit of quantification (LOQ)	89.5	59.5	89.5	59.5	146.7	77.2	146.7	77.2
Limit of blank (LOB)	–	–	17.4	14.6	–	–	29.2	29.2

### Image processing through shiny app

Our *Shiny app* includes an option to generate an HTML report of the analysis by clicking the “Download” button. The report is saved in the folder “GNSplex.gui” of our R-package. One can then move the report to a new folder and keep it as a record of the analysis. The report includes the final results as well as all relevant information, such as the data, date, applied GUI settings and package dependencies to allow reproduction. Caveat: The report generation is a dynamic procedure, where a new report automatically overrides an existing previous report.

The reports for data processing through our Shiny app are attached as supplementary information (ESM). The attached document includes the full datasets: S1_ImageJ_Calibration, S2_iPhone_ImageJ_Calibration, S3_Imager_GNSplex_calibration, S4_IPhone_GNSplex_Calibration, S5_Imager_ImageJ_Serum, S6_IPhone_ImageJ_Serum, S7_Imager_GNSplex_Serum, and S8_IPhone_GNSplex_Serum and can be downloaded.

## Conclusions

A smartphone (iPhone 5S)-based solution to compute digoxigenin concentrations from normalized intensities has been described. Either the *ImageJ* software or our open-source R-package *GNSplex* can be used for image processing. *GNSplex* can also be used to fit simple linear models to the data and by means of the fitted model, compute concentrations from normalized or standardized intensities.

A built-in Shiny app greatly increases the user-friendliness of our package and allows for extension to a web-based app that may run on a smartphone. Calibration of the lateral flow reader showed good results for reliable quantification of digoxin-spiked human serum samples. The simple cardboard darkbox can be composed in any environment with ease.

Since the quality of the acquired quantification data is comparable to readout obtained through the sophisticated laboratory hardware used as a reference, the *GNSplex* R-package and the corresponding *Shiny app* provide very attractive tools for POCTs or other lateral flow applications.

## Electronic supplementary material

ESM 1 (PDF 944 kb)
