# Supplementary material for: A smartphone readout system for gold nanoparticle-based lateral flow assays: application to monitoring of digoxigenin
Source: Mikrochim Acta. 2019 Jan 19;186(2):119. doi: 10.1007/s00604-018-3195-6 (PMC6339659; doi:10.1007/s00604-018-3195-6)
Supplement: Supplementary file 1 — (PDF 944 kb) [file 604_2018_3195_MOESM1_ESM.pdf]

Electronic Supplementary Material (ESM) on the Microchimica Acta publication entitled:

A smartphone readout system for gold nanoparticle-based lateral flow assays: application to monitoring of digoxigenin

Christoph Ruppert[a,b,c], Navneet Phogat[a,b,c], Stefan Laufer[c], Matthias Kohl[a,b] and Hans-Peter Deigner[a,b,d]

a. **Furtwangen University, Medical and Life Sciences Faculty, Jakob-Kienzle Str. 17, D-78054 Villingen-Schwenningen, Germany**
b. **Furtwangen University, Institute of Precision Medicine, Jakob-Kienzle Str. 17, D-78054 Villingen-Schwenningen, Germany**
c. **University of Tuebingen, Pharmaceutical Institute, Department of Pharmaceutical Chemistry, Auf der Morgenstelle 8, D-72076 Tuebingen, Germany**
d. **Fraunhofer Institute IZI, Leipzig, EXIM Department, Schillingallee 68, D-18057 Rostock, Germany**

The electronic supplementary includes the exported report files that can be produced with our Shiny App. It includes all normalized concentration vs. intensity graphs and additionally graphs for standardized intensity vs. concentration both with included linear fits. Additionally the assay parameters like limit of detection (LOD), limit of quantification (LOQ) and limit of blank (LOB).
The GNSplex R-Package can be downloaded from:

https://github.com/NPhogat/GNSplex

Data sets of the performed experiments are arranged as follows:

S1-Shiny app report: Imager_ImageJ_Calibration	S. 2-6	ESM
S2-Shiny app report: iPhone_ImageJ_Calibration	S. 7-11	ESM
S3-Shiny app report: Imager_GNSplex_Calibration	S. 13-16	ESM
S4-Shiny app report: iPhone_GNSplex_Calibration	S. 17-21	ESM
S5-Shiny app report: Imager_ImageJ_Serum	S. 22-26	ESM
S6-Shiny app report: iPhone_ImageJ_Serum	S. 27-31	ESM
S7-Shiny app report: Imager_GNSplex_Serum	S. 32-36	ESM
S8-Shiny app report: iPhone_GNSplex_Serum	S. 37-41	ESM

S1-Shiny app report: Imager_ImageJ_Calibration

Analysis of the data of lateral flow assay

Initial Data:

Replicate	Test	Control	Conc
R1	12819480	814577	0
R1	14326359	1185355	1
R1	13504823	1313891	20
R1	13770773	1142355	40
R1	13454238	1073477	60
R2	13875238	1391477	80
R2	13587187	787456	100
R2	13449238	1176355	NA
R2	13729652	1132477	NA
R2	12543066	972820	NA
R3	15777702	2357134	NA
R3	15684945	2172305	NA
R3	15922530	2273255	NA
R3	16134167	2437548	NA
R3	15916066	2485305	NA
R4	18939288	7613154	NA
R4	19005803	6334619	NA
R4	18720267	6102740	NA
R4	18869288	6302912	NA
R4	19328459	7455497	NA
R5	19944974	10378983	NA
R5	18514075	11656861	NA
R5	18435146	11673447	NA
R5	18430024	11652983	NA
R5	17556853	11262326	NA
R6	16017539	11280811	NA
R6	14704246	12080397	NA
R6	16332731	12576518	NA
R6	15552024	12087569	NA
R6	13211660	11650861	NA
R7	12343196	11994933	NA
R7	12865711	11579326	NA
R7	13871418	12373933	NA
R7	12513418	11912154	NA
R7	11460296	11752154	NA

Combined replicates:

	NI.crep	SI.crep	NI.sdns	SI.sdns	NI.sd	SI.sd	Concn
R1	0.0812629	12.538054	0.0120222	1.9862343	546165.3	184947.7	0
R2	0.0811499	12.735234	0.0154814	2.7457642	524290.4	226600.7	1
R3	0.1475786	6.788277	0.0069760	0.3235095	170272.6	125865.7	20
R4	0.3562058	2.828357	0.0349840	0.2672936	225384.7	712993.9	40
R5	0.6113959	1.645927	0.0510716	0.1545336	859903.2	556326.4	60
R6	0.7909906	1.271267	0.0658407	0.1058906	1251801.5	491085.8	80
R7	0.9482524	1.057357	0.0548080	0.0603023	874484.4	298300.0	100

Normalized Intensity Plot (Standardized Intensity vs Concentration):

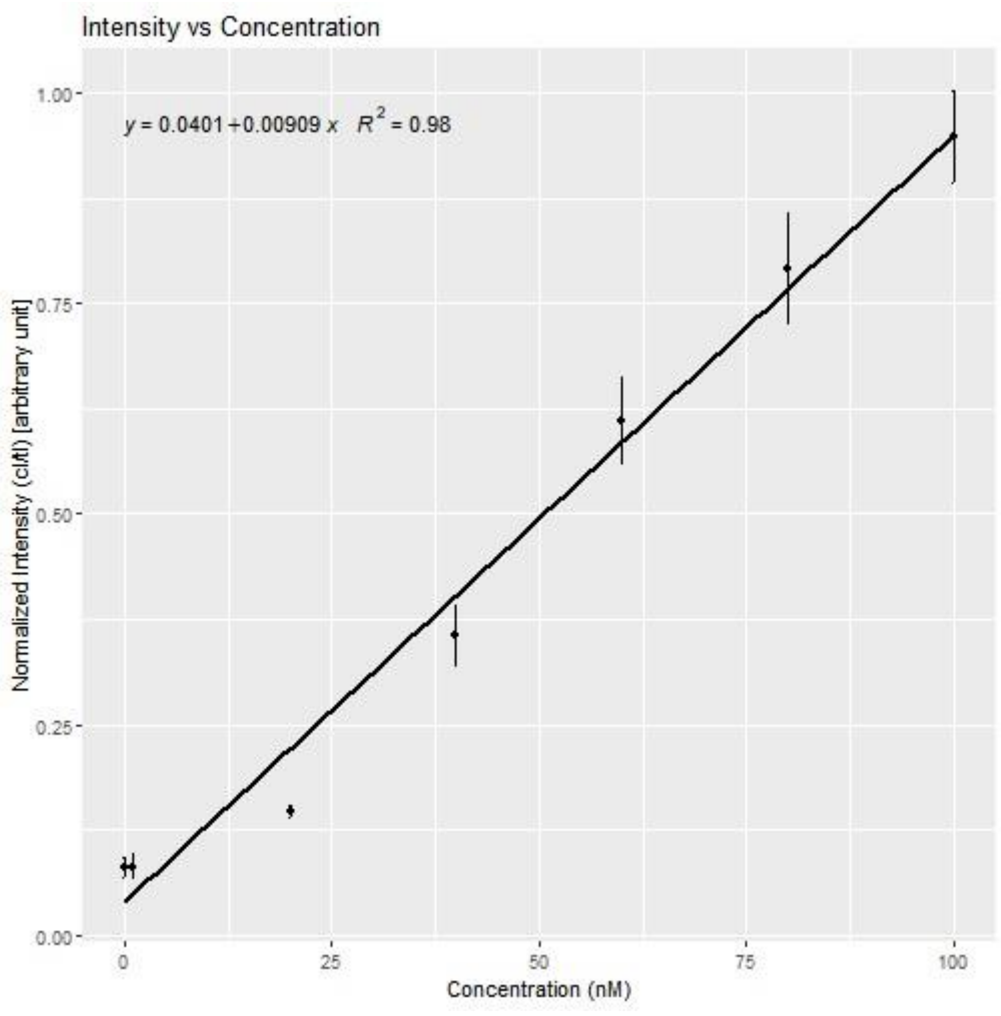

Standardized Intensity Plot (Normalized Intensity vs Concentration):

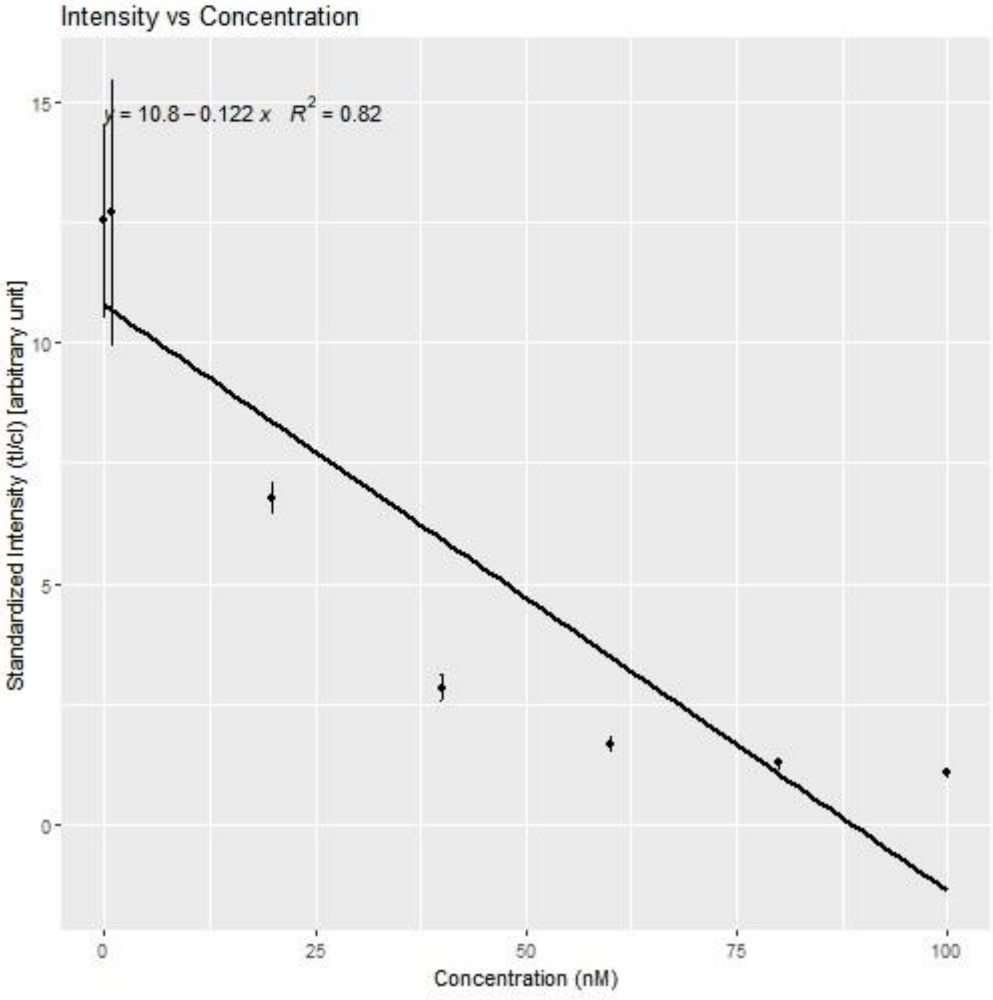

95% Confidence Interval:

	Min.Value	Max.Value
NI	0.161883	0.7000701
SI	1.610418	9.4937171

95% Confidence Interval:

	Min.Value	Max.Value
NI	0.161883	0.7000701
SI	1.610418	9.4937171

Correlation:

NI_cor	SI_cor
0.9922576	0.9076582

LOD_First Method:

lod_ni	loq_ni	lod_si	loq_si
0.1173294	0.2014846	1.238264	1.66038

LOD_Second Method:

lob_ni	lod_ni	loq_ni	lob_si	lod_si	loq_si
0.1010394	0.1265062	0.2014846	1.156555	1.330745	1.66038

Settings used during implementation

Select the type of file: .csv

Intensity value: 1

Slope value: 1

Intercept value: 1

Session Information:

R version 3.5.1 (2018-07-02) Platform: x86_64-w64-mingw32/x64 (64-bit) Running under: Windows >= 8 x64 (build 9200)

Matrix products: default

locale: [1] LC_COLLATE=English_Germany.1252 LC_CTYPE=English_Germany.1252
[3] LC_MONETARY=English_Germany.1252 LC_NUMERIC=C
[5] LC_TIME=English_Germany.1252

attached base packages: character(0)

other attached packages: [1] GNSplex_0.1.0

loaded via a namespace (and not attached): [1] tidyselect_0.2.4 locfit_1.5-9.1 purrr_0.2.5
[4] lattice_0.20-35 colorspace_1.3-2 htmltools_0.3.6
[7] yaml_2.2.0 grDevices_3.5.1 rlang_0.2.2
[10] pillar_1.3.0 later_0.7.5 glue_1.3.0
[13] withr_2.1.2 EBImage_4.22.1 BiocGenerics_0.26.0 [16] RColorBrewer_1.1-2 bindrcpp_0.2.2 jpeg_0.1-8
[19] bindr_0.1.1 plyr_1.8.4 stringr_1.3.1
[22] munsell_0.5.0 gtable_0.2.0 htmlwidgets_1.2
[25] evaluate_0.11 labeling_0.3 Biobase_2.40.0
[28] knitr_1.20 httpuv_1.4.5 parallel_3.5.1
[31] markdown_0.8 highr_0.7 methods_3.5.1
[34] Rcpp_0.12.18 xtable_1.8-3 polynom_1.3-9
[37] ggpmisc_0.3.0 scales_1.0.0 promises_1.0.1
[40] jsonlite_1.5 abind_1.4-5 mime_0.5
[43] ggplot2_3.0.0 stats_3.5.1 datasets_3.5.1

[46] graphics_3.5.1 png_0.1-7 digest_0.6.17
[49] stringi_1.1.7 tiff_0.1-5 dplyr_0.7.6
[52] shiny_1.1.0 grid_3.5.1 tools_3.5.1
[55] bitops_1.0-6 magrittr_1.5 lazyeval_0.2.1
[58] RCurl_1.95-4.11 tibble_1.4.2 crayon_1.3.4
[61] pkgconfig_2.0.2 utils_3.5.1 assertthat_0.2.0
[64] base_3.5.1 rstudioapi_0.7 R6_2.2.2
[67] fftwtools_0.9-8 compiler_3.5.1

S2-Shiny app report: iPhone_ImageJ_Calibration

Analysis of the data of lateral flow assay

Initial Data:

Replicate	Test	Control	Conc
R1	19849957	628920	0
R1	20209421	928648	1
R1	17242057	1217355	20
R1	18451936	966406	40
R1	21477271	1138648	60
R2	20175371	1168820	80
R2	17886936	877234	100
R2	20728957	1284891	NA
R2	18457421	1046062	NA
R2	22558220	1119477	NA
R3	23369563	2424276	NA
R3	26038756	2703861	NA
R3	28845605	2992589	NA
R3	23761371	2608397	NA
R3	22600886	2779518	NA
R4	30055312	11393066	NA
R4	28549664	9383045	NA
R4	31830635	9358803	NA
R4	28814342	8482510	NA
R4	25131714	9992631	NA
R5	28579271	15226945	NA
R5	29707371	22186057	NA
R5	26940785	21022208	NA
R5	26856907	19979673	NA
R5	30144664	22591137	NA
R6	26158593	22059723	NA
R6	21369643	22826894	NA
R6	24382836	23808401	NA
R6	23511785	22188430	NA
R6	22259078	22584300	NA
R7	20811744	24079380	NA
R7	19167350	23064865	NA
R7	19337886	20622208	NA
R7	17003401	21214844	NA
R7	19796643	201648029	NA

Combined replicates:

	NI.crep	SI.crep	NI.sdns	SI.sdns	NI.sd	SI.sd	Concn
R1	0.0507259	21.0886304	0.0140401	6.4622003	1636886	227763.8	0
R2	0.0550524	18.3159343	0.0055801	1.8706501	1867067	151518.8	1
R3	0.1088158	9.2299661	0.0083351	0.6552132	2539021	209968.0	20
R4	0.3387482	2.9987605	0.0478572	0.4142733	2462246	1078058.5	40
R5	0.7106566	1.4352015	0.1005035	0.2481936	1523579	2963013.6	60
R6	0.9692533	1.0382675	0.0842802	0.0943725	1865432	694530.7	80
R7	2.9720837	0.7065397	4.0332446	0.3438572	1396074	80243373.8	100

Normalized Intensity Plot (Standardized Intensity vs Concentration):

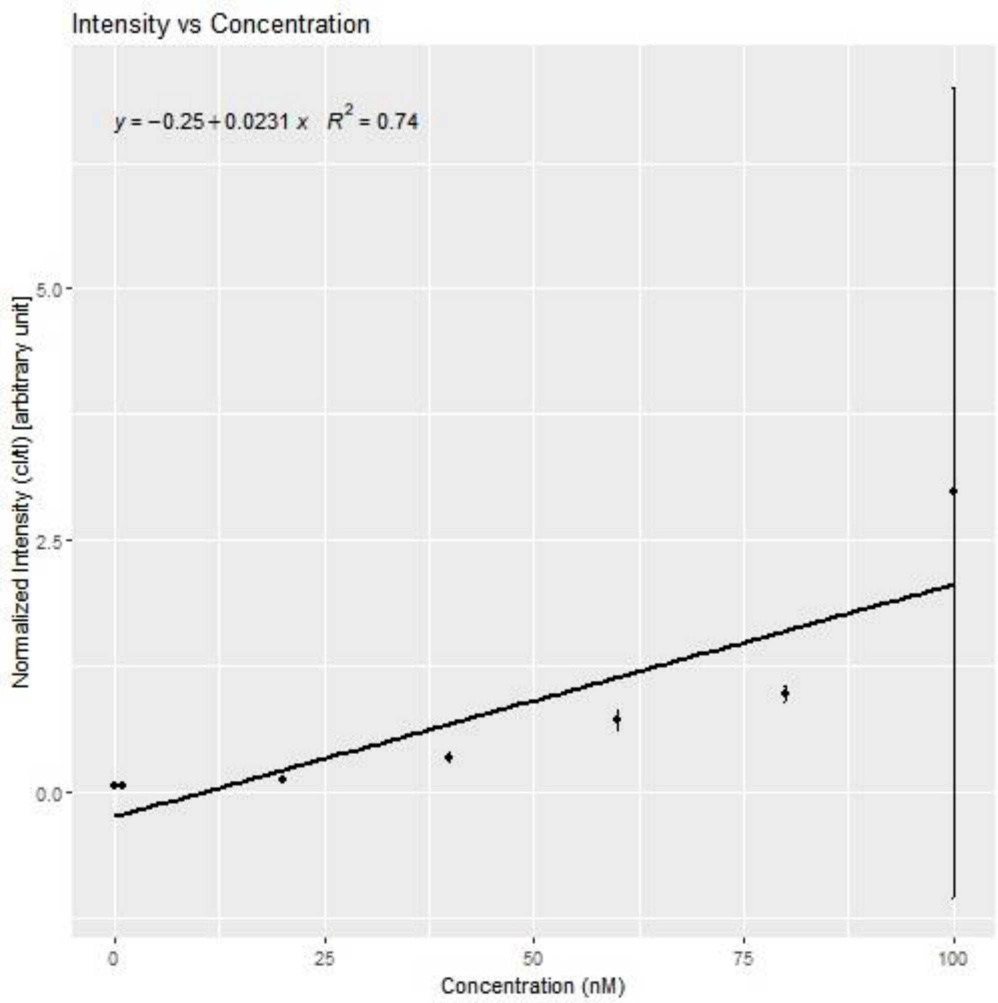

Standardized Intensity Plot (Normalized Intensity vs Concentration):

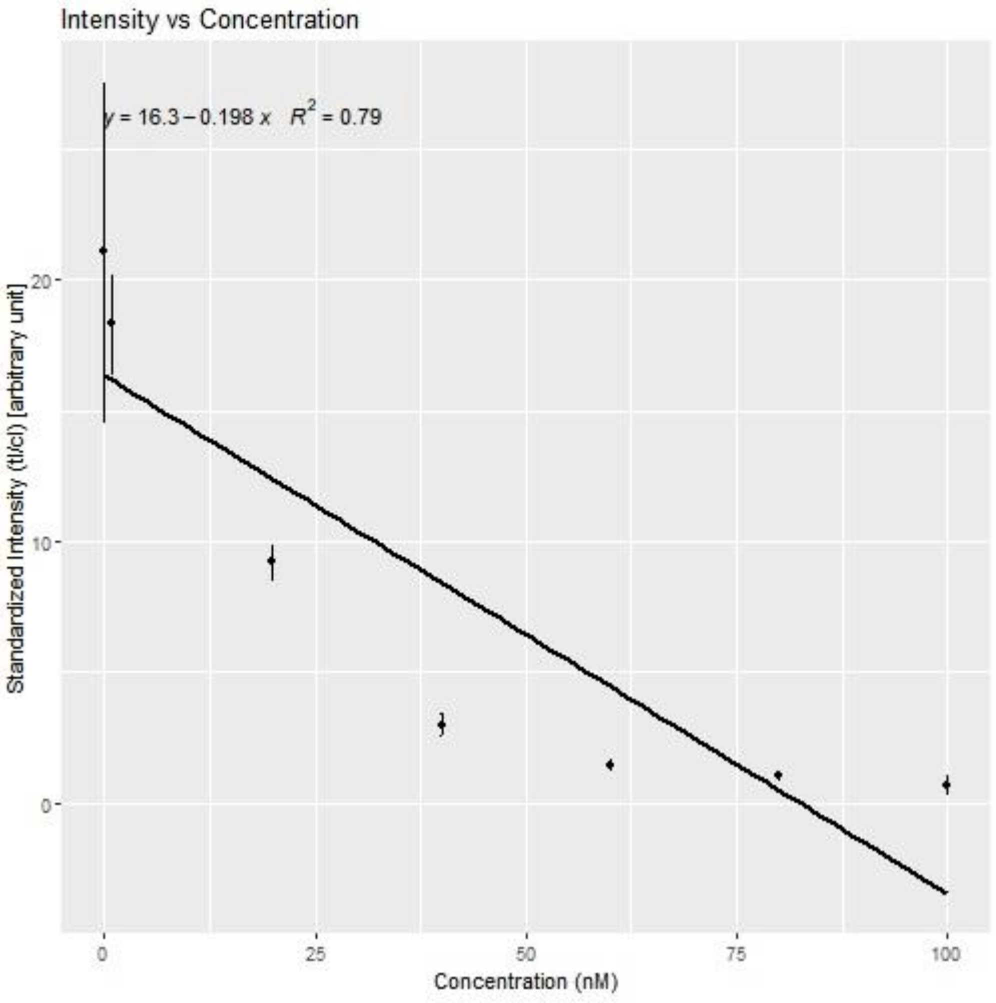

95% Confidence Interval:

Min.Value	Max.Value
-0.0455437	1.532782
1.2925845	14.368358

95% Confidence Interval:

Min.Value	Max.Value
-0.0455437	1.532782
1.2925845	14.368358

Correlation:

NI_cor	SI_cor
0.8601504	0.889901

LOD_First Method:

lod_ni	loq_ni	lod_si	loq_si
0.0928462	0.1911269	1.738111	4.145111

LOD_Second Method:

lob_ni	lod_ni	loq_ni	lob_si	lod_si	loq_si
0.0738218	0.083001	0.1911269	1.272185	1.427427	4.145111

Settings used during implementation:

Select the type of file: .csv

Intensity value: 1

Slope value: 1

Intercept value: 1

Session Information:

R version 3.5.1 (2018-07-02) Platform: x86_64-w64-mingw32/x64 (64-bit) Running under: Windows >= 8 x64 (build 9200)

Matrix products: default

locale: [1] LC_COLLATE=English_Germany.1252 LC_CTYPE=English_Germany.1252
[3] LC_MONETARY=English_Germany.1252 LC_NUMERIC=C
[5] LC_TIME=English_Germany.1252

attached base packages: character(0)

other attached packages: [1] GNSplex_0.1.0

loaded via a namespace (and not attached): [1] tidyselect_0.2.4 locfit_1.5-9.1 purrr_0.2.5
[4] lattice_0.20-35 colorspace_1.3-2 htmltools_0.3.6
[7] yaml_2.2.0 grDevices_3.5.1 rlang_0.2.2
[10] pillar_1.3.0 later_0.7.5 glue_1.3.0
[13] withr_2.1.2 EBImage_4.22.1 BiocGenerics_0.26.0 [16] RColorBrewer_1.1-2 bindrcpp_0.2.2 jpeg_0.1-8
[19] bindr_0.1.1 plyr_1.8.4 stringr_1.3.1
[22] munsell_0.5.0 gtable_0.2.0 htmlwidgets_1.2
[25] evaluate_0.11 labeling_0.3 Biobase_2.40.0
[28] knitr_1.20 httpuv_1.4.5 parallel_3.5.1
[31] markdown_0.8 highr_0.7 methods_3.5.1
[34] Rcpp_0.12.18 xtable_1.8-3 polynom_1.3-9
[37] ggpmisc_0.3.0 scales_1.0.0 promises_1.0.1

[40] jsonlite_1.5 abind_1.4-5 mime_0.5
[43] ggplot2_3.0.0 stats_3.5.1 datasets_3.5.1
[46] graphics_3.5.1 png_0.1-7 digest_0.6.17
[49] stringi_1.1.7 tiff_0.1-5 dplyr_0.7.6
[52] shiny_1.1.0 grid_3.5.1 tools_3.5.1
[55] bitops_1.0-6 magrittr_1.5 lazyeval_0.2.1
[58] RCurl_1.95-4.11 tibble_1.4.2 crayon_1.3.4
[61] pkgconfig_2.0.2 utils_3.5.1 assertthat_0.2.0
[64] base_3.5.1 rstudioapi_0.7 R6_2.2.2
[67] fftwtools_0.9-8 compiler_3.5.1

S3-Shiny app report: Imager_GNSplex_Calibration

Analysis of the data of lateral flow assay

Initial Data:

Replicate	Test	Control	Conc
R1	2.665618	1.972250	0
R1	2.686467	1.802999	1
R1	2.982301	2.274860	20
R1	2.565966	1.776844	40
R1	2.573689	1.842936	60
R2	2.430503	1.853013	80
R2	2.416658	1.709303	100
R2	2.390548	1.711391	NA
R2	2.449333	1.782119	NA
R2	2.722949	2.139932	NA
R3	2.491461	1.836071	NA
R3	2.578742	1.809790	NA
R3	2.604295	1.788031	NA
R3	2.533569	1.855411	NA
R3	2.848490	2.161237	NA
R4	2.370671	2.022635	NA
R4	2.463702	1.999698	NA
R4	2.372856	1.994070	NA
R4	2.448968	2.070735	NA
R4	2.593987	2.377702	NA
R5	2.277622	2.123299	NA
R5	2.219586	2.276303	NA
R5	2.191259	2.133412	NA
R5	2.252434	2.276235	NA
R5	2.655807	2.692046	NA
R6	2.161167	2.328301	NA
R6	2.154898	2.376353	NA
R6	1.983065	2.033739	NA
R6	2.184675	2.419267	NA
R6	2.454299	2.662822	NA
R7	2.068945	2.345260	NA
R7	2.078979	2.310772	NA
R7	1.936355	2.104124	NA
R7	2.097462	2.399156	NA
R7	2.356825	2.681556	NA

Combined replicates:

	NI.crep	SI.crep	NI.sdns	SI.sdns	NI.sd	SI.sd	Concn
R1	0.7164693	1.3986347	0.0364847	0.0712727	0.1694432	0.2048173	0
R2	0.7398159	1.3538322	0.0332087	0.0597395	0.1363896	0.1782534	1
R3	0.7232777	1.3843692	0.0288702	0.0555885	0.1394379	0.1537087	20
R4	0.8534793	1.1735368	0.0386335	0.0514672	0.0910568	0.1620143	40
R5	0.9911220	1.0101923	0.0382247	0.0400302	0.1909073	0.2311930	60
R6	1.0795999	0.9269663	0.0326476	0.0288263	0.1693662	0.2251934	80
R7	1.1226620	0.8910581	0.0235472	0.0189521	0.1531780	0.2077923	100

Normalized Intensity Plot (Standardized Intensity vs Concentration):

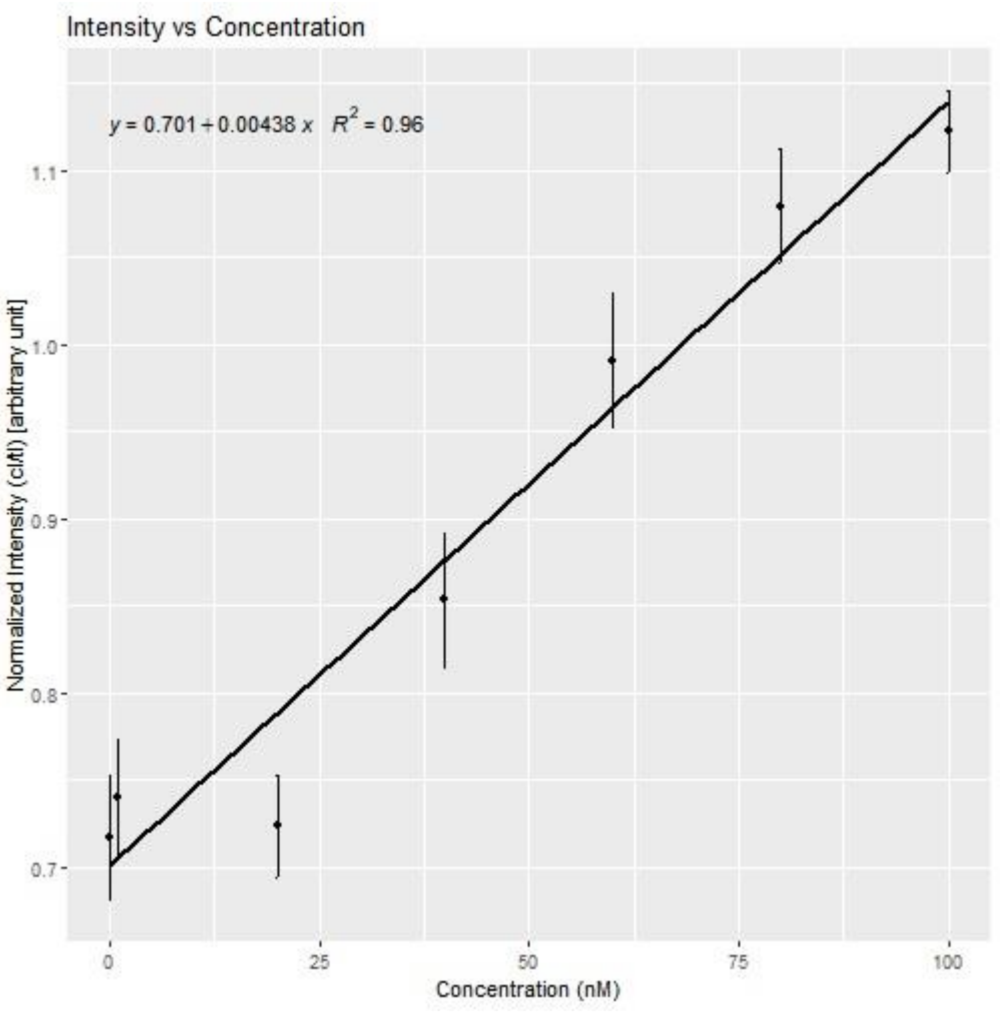

Standardized Intensity Plot (Normalized Intensity vs Concentration):

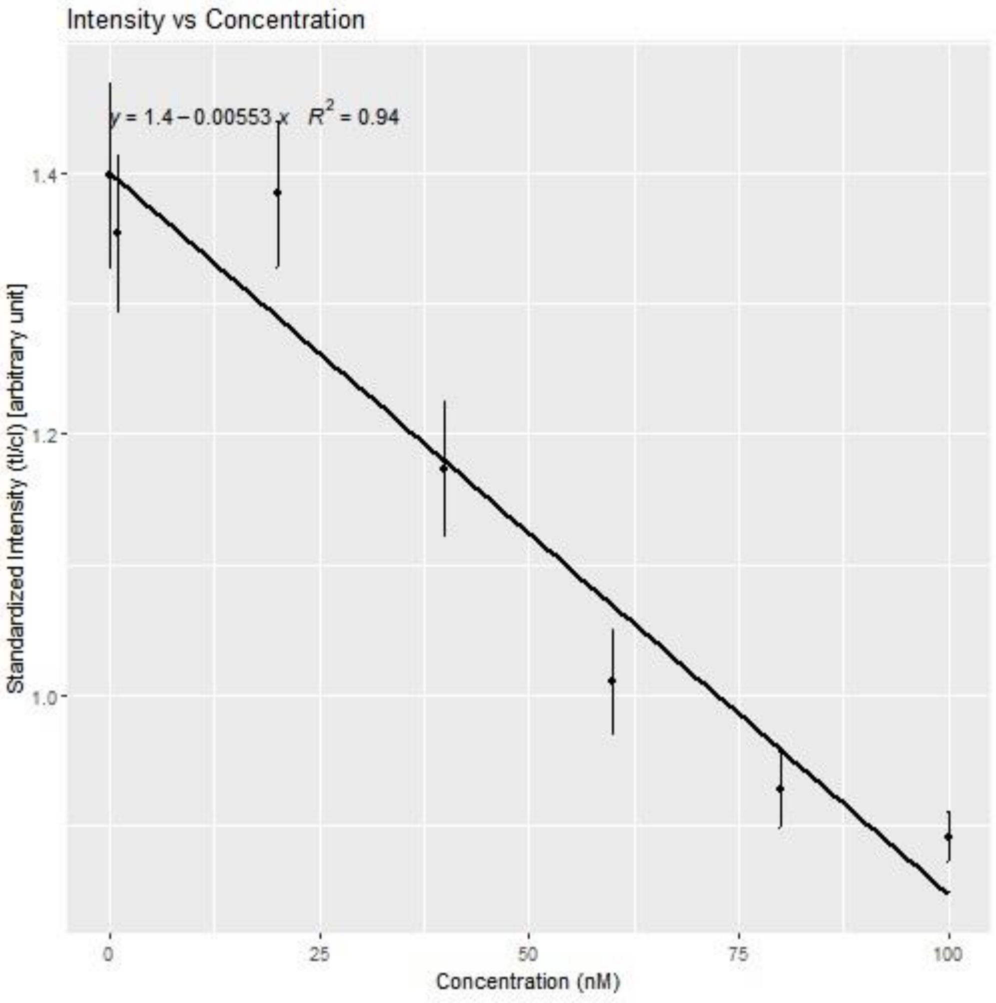

95% Confidence Interval:

Min.Value	Max.Value
0.7577965	1.021182
0.9953038	1.330008

95% Confidence Interval:

Min.Value	Max.Value
0.7577965	1.021182
0.9953038	1.330008

Correlation:

NI_cor	SI_cor
0.9777406	0.9707645

LOD_First Method:

lod_ni	loq_ni	lod_si	loq_si
0.8259236	1.081317	0.9479143	1.080579

LOD_Second Method:

lob_ni	lod_ni	loq_ni	lob_si	lod_si	loq_si
0.7764867	0.831115	1.081317	0.9222343	0.9696535	1.080579

Settings used during implementation:

Select the type of file: .txt

Intensity value: 1

Slope value: 1

Intercept value: 1

Session Information:

R version 3.5.1 (2018-07-02) Platform: x86_64-w64-mingw32/x64 (64-bit) Running under: Windows >= 8 x64 (build 9200)

Matrix products: default

locale: [1] LC_COLLATE=English_Germany.1252 LC_CTYPE=English_Germany.1252
[3] LC_MONETARY=English_Germany.1252 LC_NUMERIC=C
[5] LC_TIME=English_Germany.1252

attached base packages: character(0)

other attached packages: [1] GNSplex_0.1.0

loaded via a namespace (and not attached): [1] tidyselect_0.2.4 locfit_1.5-9.1 purrr_0.2.5
[4] lattice_0.20-35 colorspace_1.3-2 htmltools_0.3.6
[7] yaml_2.2.0 grDevices_3.5.1 rlang_0.2.2
[10] pillar_1.3.0 later_0.7.5 glue_1.3.0
[13] withr_2.1.2 EBImage_4.22.1 BiocGenerics_0.26.0 [16] RColorBrewer_1.1-2 bindrcpp_0.2.2 jpeg_0.1-8
[19] bindr_0.1.1 plyr_1.8.4 stringr_1.3.1
[22] munsell_0.5.0 gtable_0.2.0 htmlwidgets_1.2
[25] evaluate_0.11 labeling_0.3 Biobase_2.40.0
[28] knitr_1.20 httpuv_1.4.5 parallel_3.5.1
[31] markdown_0.8 highr_0.7 methods_3.5.1
[34] Rcpp_0.12.18 xtable_1.8-3 polynom_1.3-9
[37] ggpmisc_0.3.0 scales_1.0.0 promises_1.0.1
[40] jsonlite_1.5 abind_1.4-5 mime_0.5
[43] ggplot2_3.0.0 stats_3.5.1 datasets_3.5.1
[46] graphics_3.5.1 png_0.1-7 digest_0.6.17
[49] stringi_1.1.7 tiff_0.1-5 dplyr_0.7.6

[52] shiny_1.1.0 grid_3.5.1 tools_3.5.1
[55] bitops_1.0-6 magrittr_1.5 lazyeval_0.2.1
[58] RCurl_1.95-4.11 tibble_1.4.2 crayon_1.3.4
[61] pkgconfig_2.0.2 utils_3.5.1 assertthat_0.2.0
[64] base_3.5.1 rstudioapi_0.7 R6_2.2.2
[67] fftwtools_0.9-8 compiler_3.5.1

S4-Shiny app report: iPhone_GNSplex_Calibration

Analysis of the data of lateral flow assay

Initial Data:

Replicate	Test	Control	Conc
R1	2.253461	1.667461	0
R1	2.253461	1.666799	1
R1	2.040317	1.636620	20
R1	2.115741	1.664692	40
R1	2.050332	1.664525	60
R2	2.063235	1.652308	80
R2	2.068786	1.633346	100
R2	2.107026	1.639472	NA
R2	2.130163	1.670773	NA
R2	1.894965	1.604337	NA
R3	2.009606	1.656089	NA
R3	1.997425	1.671873	NA
R3	1.913738	1.624843	NA
R3	1.995282	1.668776	NA
R3	1.939495	1.653739	NA
R4	1.901865	1.752297	NA
R4	1.978616	1.789533	NA
R4	1.928561	1.730834	NA
R4	1.899058	1.733774	NA
R4	1.874816	1.750269	NA
R5	1.877766	1.809685	NA
R5	1.783899	1.776576	NA
R5	1.809998	1.793537	NA
R5	1.820237	1.810064	NA
R5	1.808478	1.802381	NA
R6	1.735298	1.760593	NA
R6	1.787728	1.817140	NA
R6	1.747525	1.759287	NA
R6	1.824096	1.849292	NA
R6	1.739770	1.760294	NA
R7	1.735752	1.770822	NA
R7	1.795284	1.823320	NA
R7	1.751836	1.762549	NA
R7	1.714216	1.781534	NA
R7	1.805152	1.873045	NA

Combined replicates:

	NI.crep	SI.crep	NI.sdns	SI.sdns	NI.sd	SI.sd	Concn
R1	0.7760804	1.2905599	0.0342921	0.0575385	0.1052104	0.0131435	0
R2	0.7998845	1.2513173	0.0274313	0.0414408	0.0924687	0.0245706	1
R3	0.8398336	1.1908873	0.0113733	0.0161701	0.0419814	0.0186191	20
R4	0.9139604	1.0943489	0.0141657	0.0169053	0.0395594	0.0233978	40
R5	0.9883168	1.0119847	0.0139122	0.0145033	0.0349035	0.0139568	60
R6	1.0126739	0.9874954	0.0037177	0.0036349	0.0381291	0.0416543	80
R7	1.0237635	0.9769414	0.0143415	0.0136821	0.0388376	0.0459590	100

Normalized Intensity Plot (Standardized Intensity vs Concentration):

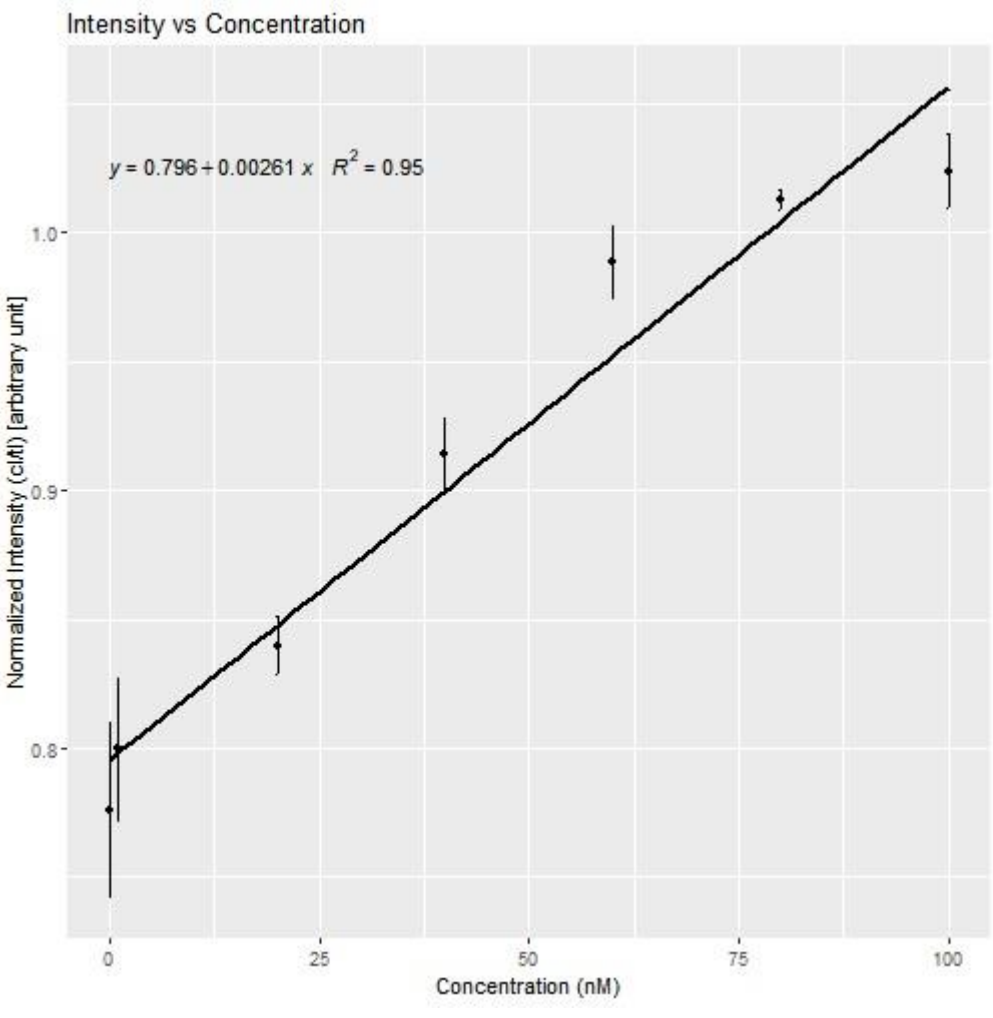

Standardized Intensity Plot (Normalized Intensity vs Concentration):

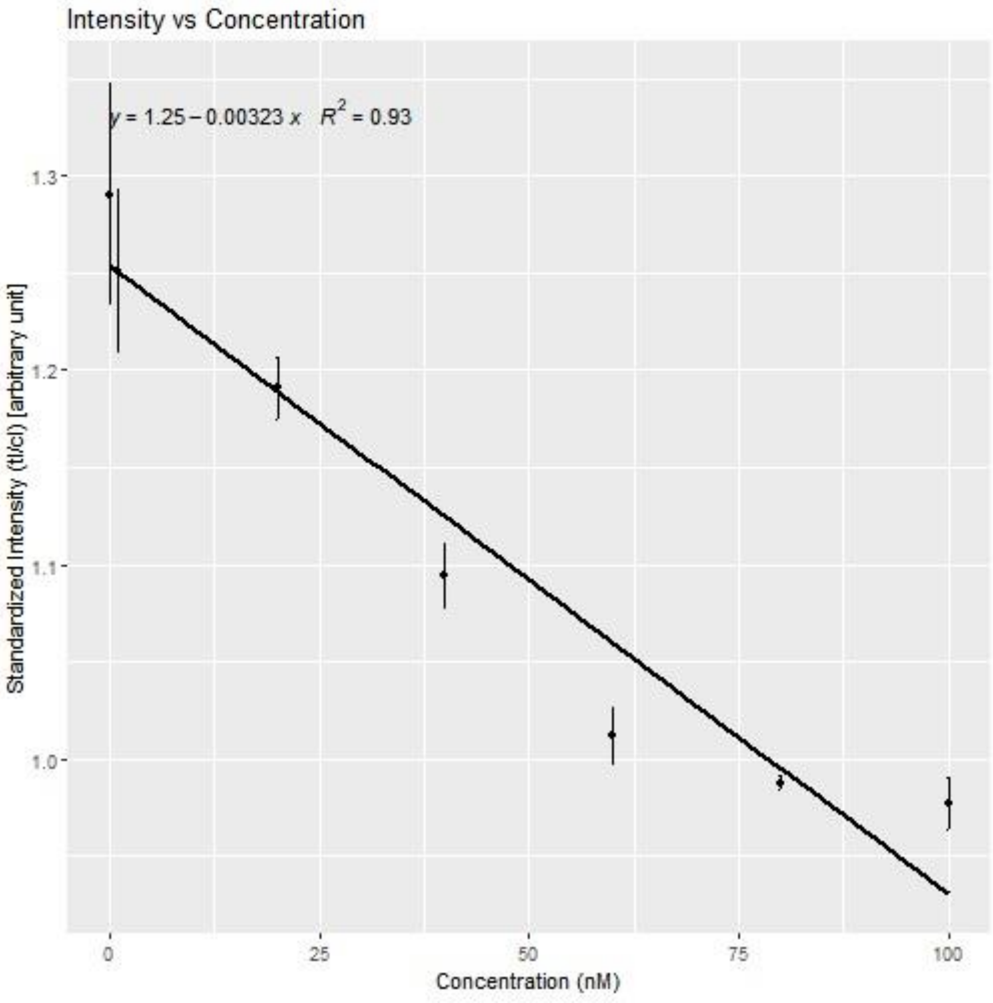

95% Confidence Interval:

**Min.Value**	**Max.Value**
0.8293613	0.9862139
1.0164626	1.2131188

95% Confidence Interval:

**Min.Value**	**Max.Value**
0.8293613	0.9862139
1.0164626	1.2131188

Correlation:

**NI_cor**	**SI_cor**
0.9757593	0.9660987

LOD_First Method:

lod_ni	loq_ni	lod_si	loq_si
0.8789566	1.119001	1.017988	1.113762

LOD_Second Method:

lob_ni	lod_ni	loq_ni	lob_si	lod_si	loq_si
0.8324909	0.8776154	1.119001	0.9994485	1.005428	1.113762

Settings used during implementation:

Select the type of file: .txt

Intensity value: 1

Slope value: 1

Intercept value: 1

Session Information:

R version 3.5.1 (2018-07-02) Platform: x86_64-w64-mingw32/x64 (64-bit) Running under: Windows >= 8 x64 (build 9200)

Matrix products: default

locale: [1] LC_COLLATE=English_Germany.1252 LC_CTYPE=English_Germany.1252
[3] LC_MONETARY=English_Germany.1252 LC_NUMERIC=C
[5] LC_TIME=English_Germany.1252

attached base packages: character(0)

other attached packages: [1] GNSplex_0.1.0

loaded via a namespace (and not attached): [1] tidyselect_0.2.4 locfit_1.5-9.1 purrr_0.2.5
[4] lattice_0.20-35 colorspace_1.3-2 htmltools_0.3.6
[7] yaml_2.2.0 grDevices_3.5.1 rlang_0.2.2
[10] pillar_1.3.0 later_0.7.5 glue_1.3.0
[13] withr_2.1.2 EBImage_4.22.1 BiocGenerics_0.26.0 [16] RColorBrewer_1.1-2 bindrcpp_0.2.2 jpeg_0.1-8
[19] bindr_0.1.1 plyr_1.8.4 stringr_1.3.1
[22] munsell_0.5.0 gtable_0.2.0 htmlwidgets_1.2
[25] evaluate_0.11 labeling_0.3 Biobase_2.40.0
[28] knitr_1.20 httpuv_1.4.5 parallel_3.5.1
[31] markdown_0.8 highr_0.7 methods_3.5.1
[34] Rcpp_0.12.18 xtable_1.8-3 polynom_1.3-9
[37] ggpmisc_0.3.0 scales_1.0.0 promises_1.0.1
[40] jsonlite_1.5 abind_1.4-5 mime_0.5
[43] ggplot2_3.0.0 stats_3.5.1 datasets_3.5.1

[46] graphics_3.5.1 png_0.1-7 digest_0.6.17
[49] stringi_1.1.7 tiff_0.1-5 dplyr_0.7.6
[52] shiny_1.1.0 grid_3.5.1 tools_3.5.1
[55] bitops_1.0-6 magrittr_1.5 lazyeval_0.2.1
[58] RCurl_1.95-4.11 tibble_1.4.2 crayon_1.3.4
[61] pkgconfig_2.0.2 utils_3.5.1 assertthat_0.2.0
[64] base_3.5.1 rstudioapi_0.7 R6_2.2.2
[67] fftwtools_0.9-8 compiler_3.5.1

S5-Shiny app report: Imager_ImageJ_Serum

Analysis of the data of lateral flow assay

Initial Data:

Replicate	Control	Test	Conc
R1	3.304790	12.57558	0
R1	2.927962	11.61563	1
R1	2.324374	12.88241	5
R2	3.209134	12.51863	10
R2	3.168548	12.72105	15
R2	3.047426	12.63887	20
R3	2.284255	13.39434	25
R3	3.421669	12.93004	30
R3	2.040255	12.24482	40
R4	3.581790	13.05705	60
R4	3.621841	13.42092	80
R4	3.833912	12.87734	100
R5	3.615962	13.36729	NA
R5	3.789912	13.47992	NA
R5	3.856255	13.69051	NA
R6	4.274548	14.03180	NA
R6	4.015669	13.97092	NA
R6	4.341790	14.02622	NA
R7	4.783497	14.45309	NA
R7	4.814033	14.68563	NA
R7	4.843083	14.40132	NA
R8	5.939205	15.14144	NA
R8	4.408740	15.82995	NA
R8	5.440497	14.10468	NA
R9	7.009740	15.16856	NA
R9	6.647447	15.42109	NA
R9	6.320083	14.92415	NA
R10	9.058447	15.54256	NA
R10	8.502397	15.04863	NA
R10	8.208447	15.78887	NA
R11	10.360276	14.71515	NA
R11	9.652397	15.13817	NA
R11	10.073690	15.22595	NA
R12	10.443861	13.54049	NA
R12	9.145225	16.54265	NA
R12	10.262640	13.51956	NA

Combined replicates:

	NI.crep	SI.crep	NI.sdns	SI.sdns	NI.sd	SI.sd	Concn
R1	0.2317650	4.438237	0.0447795	0.9595780	0.4945593	0.6608547	0
R2	0.2488477	4.021039	0.0076193	0.1233468	0.0841301	0.1018020	1
R3	0.2005967	5.214750	0.0554885	1.2454169	0.7372868	0.5782851	5
R4	0.2806364	3.569916	0.0149661	0.1852907	0.1354890	0.2769441	10
R5	0.2777781	3.601248	0.0063012	0.0827679	0.1240972	0.1640659	15
R6	0.3005372	3.330753	0.0116138	0.1310918	0.1721892	0.0336521	20
R7	0.3316890	3.015207	0.0042902	0.0388793	0.0297961	0.1514312	25
R8	0.3521592	2.910841	0.0638686	0.5890683	0.7805506	0.8684703	30
R9	0.4388884	2.281721	0.0204757	0.1041054	0.3449759	0.2484856	40
R10	0.5558995	1.803076	0.0324347	0.1077362	0.4316824	0.3769607	60
R11	0.6677628	1.500044	0.0336419	0.0746519	0.3560693	0.2731219	80
R12	0.6944096	1.474248	0.1227660	0.2899911	0.7033151	1.7393728	100

Normalized Intensity Plot (Standardized Intensity vs Concentration):

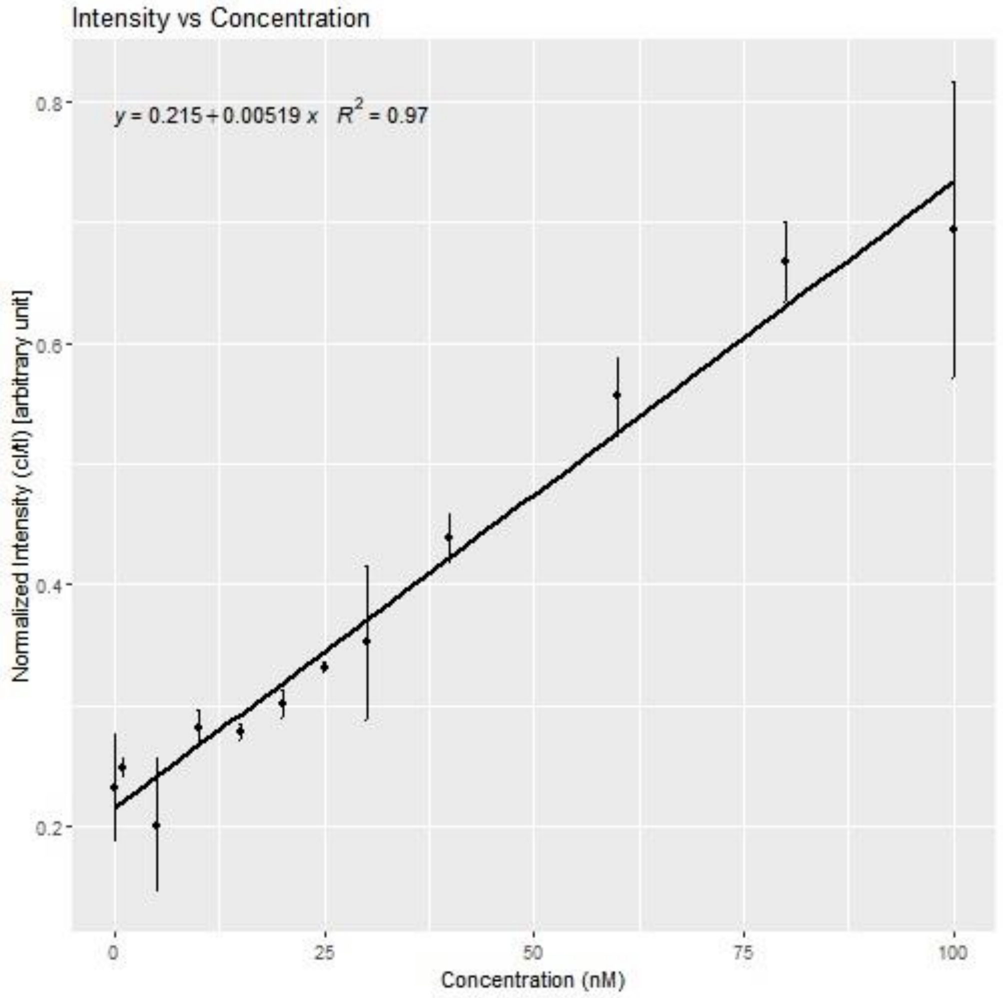

Standardized Intensity Plot (Normalized Intensity vs Concentration):

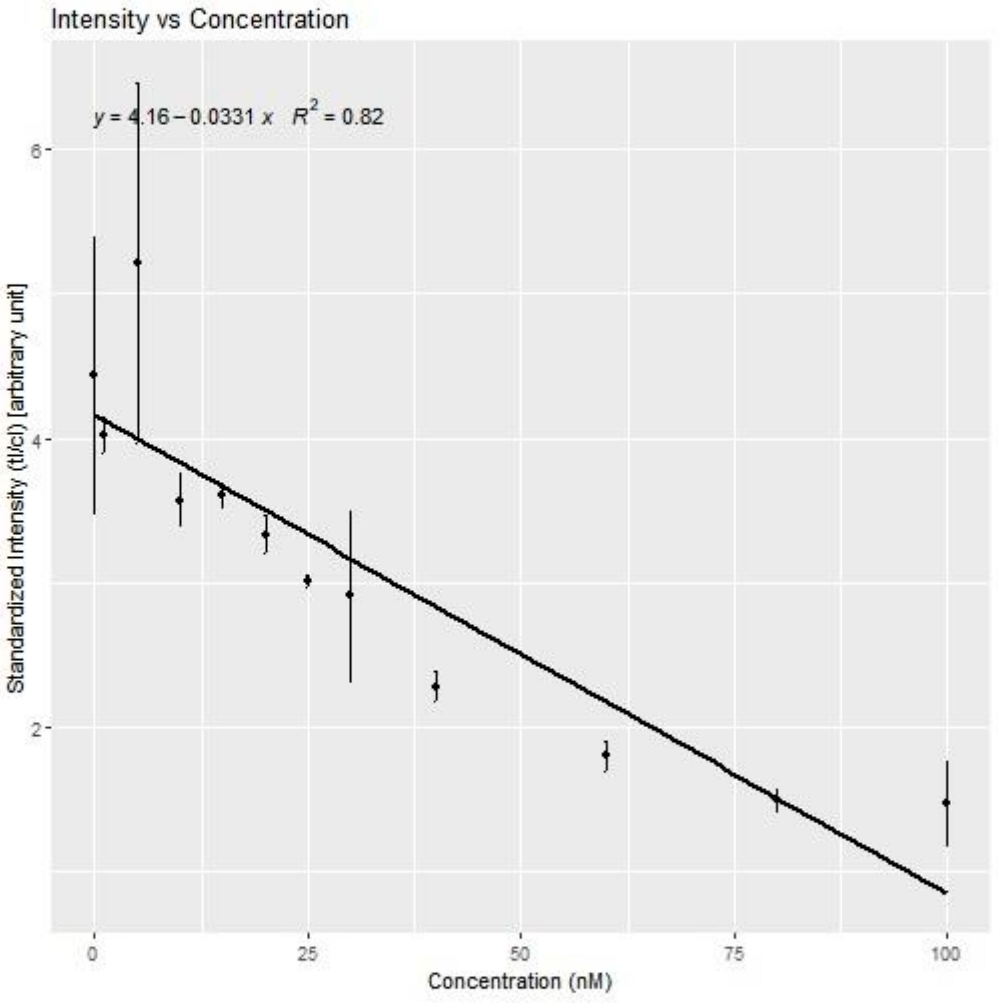

95% Confidence Interval:

**Min.Value**	**Max.Value**
0.2837155	0.4797794
2.4173174	3.7761959

95% Confidence Interval:

**Min.Value**	**Max.Value**
0.2837155	0.4797794
2.4173174	3.7761959

Correlation:

**NI_cor**	**SI_cor**
0.9871345	0.9078776

LOD_First Method:

lod_ni	loq_ni	lod_si	loq_si
0.3661037	0.6795605	2.344221	4.374159

LOD_Second Method:

lob_ni	lod_ni	loq_ni	lob_si	lod_si	loq_si
0.3054274	0.3179611	0.6795605	1.951283	2.074085	4.374159

Settings used during implementation:

Select the type of file: .txt

Intensity value: 1

Slope value: 1

Intercept value: 1

Session Information:

R version 3.5.1 (2018-07-02) Platform: x86_64-w64-mingw32/x64 (64-bit) Running under: Windows >= 8 x64 (build 9200)

Matrix products: default

locale: [1] LC_COLLATE=English_Germany.1252 LC_CTYPE=English_Germany.1252
[3] LC_MONETARY=English_Germany.1252 LC_NUMERIC=C
[5] LC_TIME=English_Germany.1252

attached base packages: character(0)

other attached packages: [1] GNSplex_0.1.0

loaded via a namespace (and not attached): [1] tidyselect_0.2.4 locfit_1.5-9.1 purrr_0.2.5
[4] lattice_0.20-35 colorspace_1.3-2 htmltools_0.3.6
[7] yaml_2.2.0 grDevices_3.5.1 rlang_0.2.2
[10] pillar_1.3.0 later_0.7.5 glue_1.3.0
[13] withr_2.1.2 EBImage_4.22.1 BiocGenerics_0.26.0 [16] RColorBrewer_1.1-2 bindrcpp_0.2.2 jpeg_0.1-8
[19] bindr_0.1.1 plyr_1.8.4 stringr_1.3.1
[22] munsell_0.5.0 gtable_0.2.0 htmlwidgets_1.2
[25] evaluate_0.11 labeling_0.3 Biobase_2.40.0
[28] knitr_1.20 httpuv_1.4.5 parallel_3.5.1
[31] markdown_0.8 highr_0.7 methods_3.5.1
[34] Rcpp_0.12.18 xtable_1.8-3 polynom_1.3-9
[37] ggpmisc_0.3.0 scales_1.0.0 promises_1.0.1
[40] jsonlite_1.5 abind_1.4-5 mime_0.5
[43] ggplot2_3.0.0 stats_3.5.1 datasets_3.5.1
[46] graphics_3.5.1 png_0.1-7 digest_0.6.17
[49] stringi_1.1.7 tiff_0.1-5 dplyr_0.7.6

[52] shiny_1.1.0 grid_3.5.1 tools_3.5.1
[55] bitops_1.0-6 magrittr_1.5 lazyeval_0.2.1
[58] RCurl_1.95-4.11 tibble_1.4.2 crayon_1.3.4
[61] pkgconfig_2.0.2 utils_3.5.1 assertthat_0.2.0
[64] base_3.5.1 rstudioapi_0.7 R6_2.2.2
[67] fftwtools_0.9-8 compiler_3.5.1

S6-Shiny app report: iPhone_ImageJ_Serum

Analysis of the data of lateral flow assay

Initial Data:

Replicate	Control	Test	Conc
R1	3.655782	18.23130	0
R1	2.723397	15.03921	1
R1	2.238912	19.25371	5
R2	3.249296	16.32562	10
R2	3.340175	17.10187	15
R2	3.008933	16.99386	20
R3	2.806054	20.21449	25
R3	3.629589	17.90752	30
R3	2.102740	18.41913	40
R4	3.405054	17.68823	60
R4	4.081180	19.50425	80
R4	4.078539	18.41547	100
R5	4.197044	19.65837	NA
R5	4.059418	18.33676	NA
R5	4.437953	19.20935	NA
R6	4.971660	18.93552	NA
R6	4.446953	18.47677	NA
R6	4.951368	20.44071	NA
R7	5.718853	19.27906	NA
R7	5.731489	20.85752	NA
R7	6.069196	22.00054	NA
R8	8.272924	24.29020	NA
R8	5.319196	23.12944	NA
R8	5.757125	17.42850	NA
R9	9.160681	20.03211	NA
R9	8.568974	19.74645	NA
R9	9.643167	22.69484	NA
R10	15.171359	23.87086	NA
R10	15.102894	23.21654	NA
R10	14.302702	23.22196	NA
R11	17.204823	21.00291	NA
R11	16.489409	21.02323	NA
R11	18.676359	22.04011	NA
R12	21.782673	19.89194	NA
R12	15.729409	24.47032	NA
R12	20.421966	19.25157	NA

Combined replicates:

	NI.crep	SI.crep	NI.sdns	SI.sdns	NI.sd	SI.sd	Concn
R1	0.1659645	6.369595	0.0441078	1.9496828	0.7201375	2.1983646	0
R2	0.1904670	5.264070	0.0117589	0.3357500	0.1711503	0.4204692	1
R3	0.1518866	6.965743	0.0456872	1.9239974	0.7642129	1.2115543	5
R4	0.2077410	4.829661	0.0145433	0.3425561	0.3896014	0.9139887	10
R5	0.2219704	4.509793	0.0087807	0.1778300	0.1916015	0.6720202	15
R6	0.2484887	4.030638	0.0122085	0.1926719	0.2972551	1.0273880	20
R7	0.2824312	3.545068	0.0123130	0.1507916	0.1987234	1.3665367	25
R8	0.3002967	3.437232	0.0611159	0.7903209	1.5940272	3.6726693	30
R9	0.4387186	2.281541	0.0167152	0.0856780	0.5380211	1.6260737	40
R10	0.6339986	1.578082	0.0173578	0.0433794	0.4829699	0.3762146	60
R11	0.8169623	1.225273	0.0315767	0.0475840	1.1150475	0.5930489	80
R12	0.9328802	1.137198	0.2518040	0.3627374	3.1757839	2.8462553	100

Normalized Intensity Plot (Standardized Intensity vs Concentration):

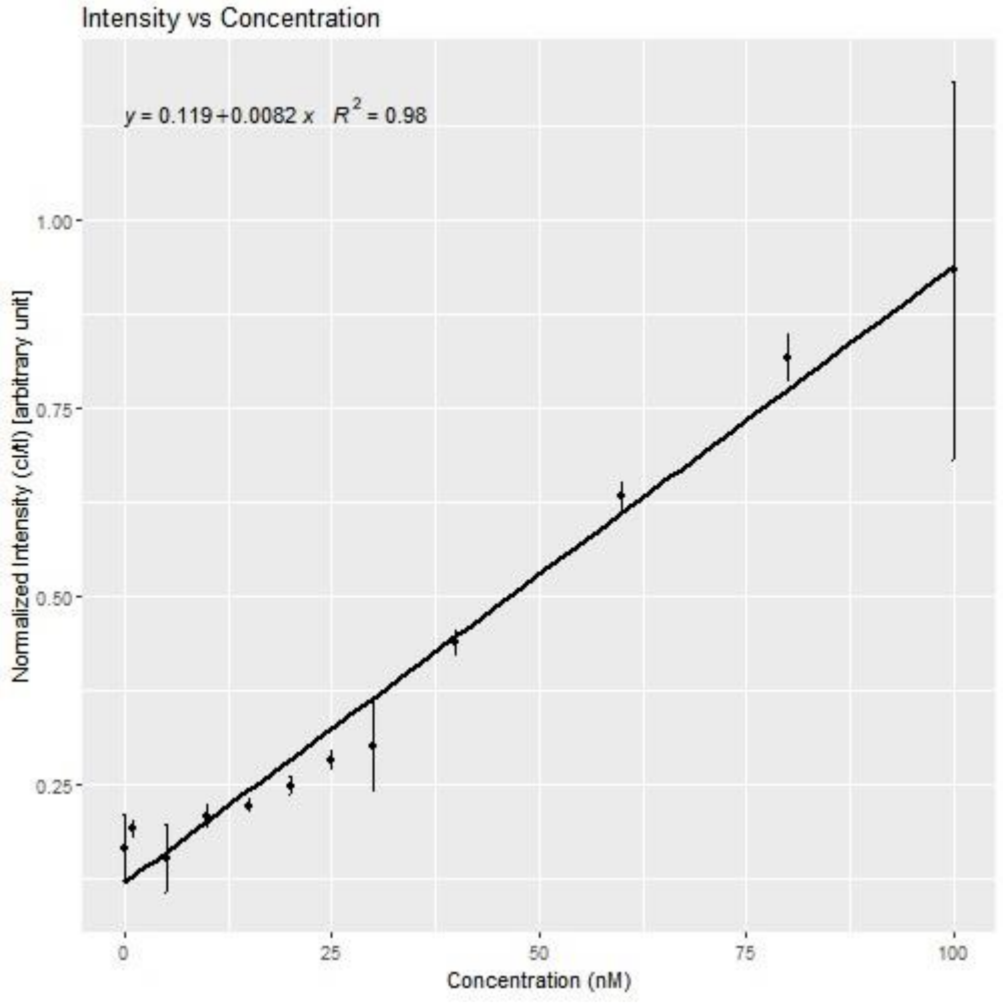

Standardized Intensity Plot (Normalized Intensity vs Concentration):

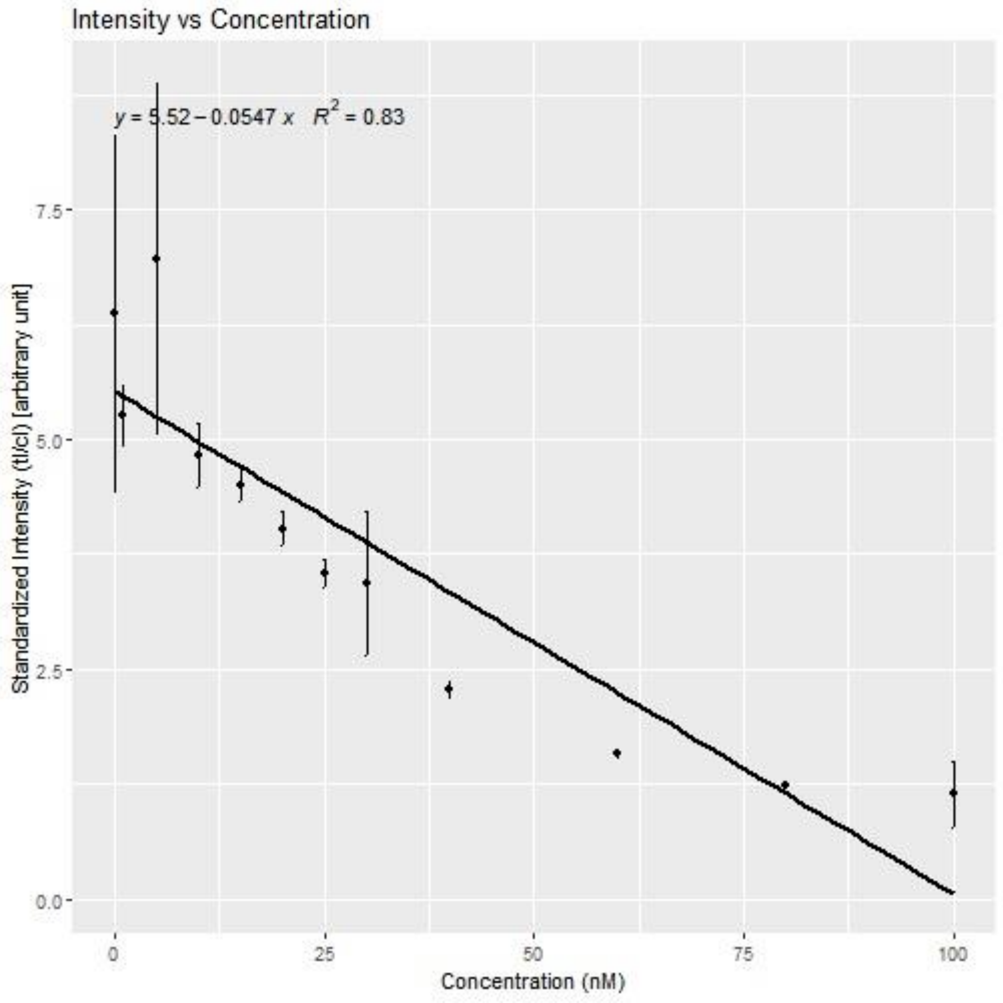

95% Confidence Interval:

**Min.Value**	**Max.Value**
0.2283117	0.5369893
2.6426789	4.8863038

95% Confidence Interval:

**Min.Value**	**Max.Value**
0.2283117	0.5369893
2.6426789	4.8863038

Correlation:

**NI_cor**	**SI_cor**
0.989628	0.9088938

LOD_First Method:

lod_ni	loq_ni	lod_si	loq_si
0.2982879	0.6070426	2.22541	4.764572

LOD_Second Method:

lob_ni	lod_ni	loq_ni	lob_si	lod_si	loq_si
0.2385218	0.2578652	0.6070426	1.733901	1.812177	4.764572

Settings used during implementation:

Select the type of file: .txt

Intensity value: 1

Slope value: 1

Intercept value: 1

Session Information:

R version 3.5.1 (2018-07-02) Platform: x86_64-w64-mingw32/x64 (64-bit) Running under: Windows >= 8 x64 (build 9200)

Matrix products: default

locale: [1] LC_COLLATE=English_Germany.1252 LC_CTYPE=English_Germany.1252
[3] LC_MONETARY=English_Germany.1252 LC_NUMERIC=C
[5] LC_TIME=English_Germany.1252

attached base packages: character(0)

other attached packages: [1] GNSplex_0.1.0

loaded via a namespace (and not attached): [1] tidyselect_0.2.4 locfit_1.5-9.1 purrr_0.2.5
[4] lattice_0.20-35 colorspace_1.3-2 htmltools_0.3.6
[7] yaml_2.2.0 grDevices_3.5.1 rlang_0.2.2
[10] pillar_1.3.0 later_0.7.5 glue_1.3.0
[13] withr_2.1.2 EBImage_4.22.1 BiocGenerics_0.26.0 [16] RColorBrewer_1.1-2 bindrcpp_0.2.2 jpeg_0.1-8
[19] bindr_0.1.1 plyr_1.8.4 stringr_1.3.1
[22] munsell_0.5.0 gtable_0.2.0 htmlwidgets_1.2
[25] evaluate_0.11 labeling_0.3 Biobase_2.40.0
[28] knitr_1.20 httpuv_1.4.5 parallel_3.5.1
[31] markdown_0.8 highr_0.7 methods_3.5.1
[34] Rcpp_0.12.18 xtable_1.8-3 polynom_1.3-9
[37] ggpmisc_0.3.0 scales_1.0.0 promises_1.0.1
[40] jsonlite_1.5 abind_1.4-5 mime_0.5
[43] ggplot2_3.0.0 stats_3.5.1 datasets_3.5.1

[46] graphics_3.5.1 png_0.1-7 digest_0.6.17
[49] stringi_1.1.7 tiff_0.1-5 dplyr_0.7.6
[52] shiny_1.1.0 grid_3.5.1 tools_3.5.1
[55] bitops_1.0-6 magrittr_1.5 lazyeval_0.2.1
[58] RCurl_1.95-4.11 tibble_1.4.2 crayon_1.3.4
[61] pkgconfig_2.0.2 utils_3.5.1 assertthat_0.2.0
[64] base_3.5.1 rstudioapi_0.7 R6_2.2.2
[67] fftwtools_0.9-8 compiler_3.5.1

S7-Shiny app report: Imager_GNSplex_Serum

Analysis of the data of lateral flow assay

Initial Data:

Replicate	Test	Control	Conc
R1	2.495087	2.192661	0
R1	2.709717	2.193412	1
R1	2.698113	2.231806	5
R2	2.508678	2.114448	10
R2	2.713118	2.194251	15
R2	2.677233	2.290130	20
R3	2.544692	2.075266	25
R3	2.617144	2.236919	30
R3	2.818819	2.197077	40
R4	2.600412	2.101003	60
R4	2.554399	2.204841	80
R4	2.555401	2.316758	100
R5	2.627631	2.179235	NA
R5	2.543842	2.259638	NA
R5	2.650721	2.239813	NA
R6	2.467461	2.218733	NA
R6	2.641235	2.275245	NA
R6	2.678383	2.307905	NA
R7	2.533235	2.248420	NA
R7	2.765401	2.332045	NA
R7	2.673531	2.315511	NA
R8	2.475173	2.224196	NA
R8	2.690972	2.325040	NA
R8	2.639707	2.444197	NA
R9	2.447104	2.292898	NA
R9	2.755486	2.531030	NA
R9	2.693439	2.414114	NA
R10	2.333501	2.343541	NA
R10	2.600692	2.547074	NA
R10	2.569431	2.592836	NA
R11	2.278629	2.409436	NA
R11	2.467937	2.575594	NA
R11	2.620096	2.692390	NA
R12	2.328753	2.478548	NA
R12	2.527264	2.747437	NA
R12	2.473426	2.571805	NA

Combined replicates:

	NI.crep	SI.crep	NI.sdns	SI.sdns	NI.sd	SI.sd	Concn
R1	0.8384753	1.1940842	0.0360203	0.0504003	0.1207064	0.0223867	0
R2	0.8356730	1.1973145	0.0241412	0.0350069	0.1091591	0.0879635	1
R3	0.8165589	1.2263878	0.0376535	0.0565047	0.1420491	0.0842190	5
R4	0.8592390	1.1664162	0.0494475	0.0676909	0.0262811	0.1079027	10
R5	0.8542046	1.1716630	0.0305254	0.0412757	0.0562389	0.0418875	15
R6	0.8741025	1.1444956	0.0217326	0.0280527	0.1125948	0.0451145	20
R7	0.8656498	1.1557060	0.0221408	0.0295916	0.1169217	0.0442864	25
R8	0.8961840	1.1167388	0.0310308	0.0388460	0.1127451	0.1101275	30
R9	0.9172736	1.0905468	0.0203746	0.0242794	0.1631105	0.1190725	40
R10	0.9975982	1.0025800	0.0159567	0.0161710	0.1460772	0.1327076	60
R11	1.0428735	0.9590201	0.0149210	0.0137374	0.1710700	0.1421928	80
R12	1.0637392	0.9403910	0.0236778	0.0209546	0.1026608	0.1365313	100

Normalized Intensity Plot (Standardized Intensity vs Concentration):

Intensity vs Concentration

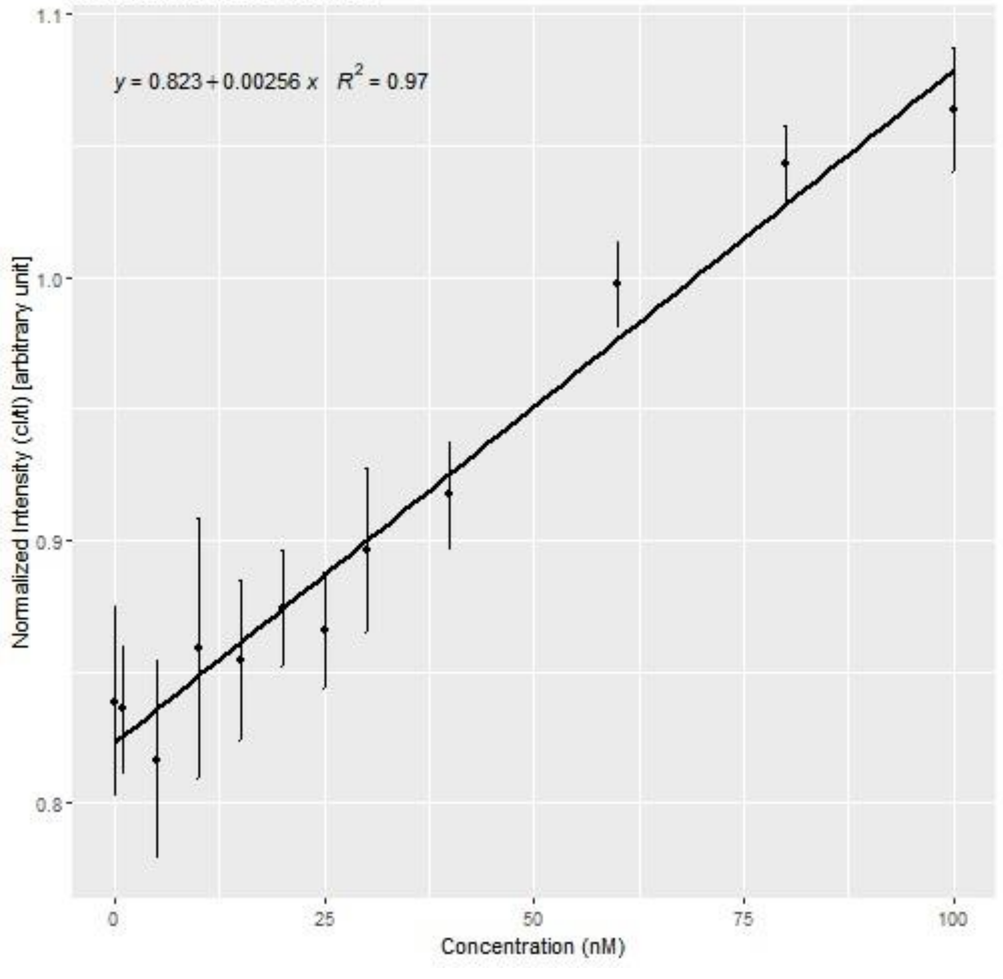

Standardized Intensity Plot (Normalized Intensity vs Concentration):

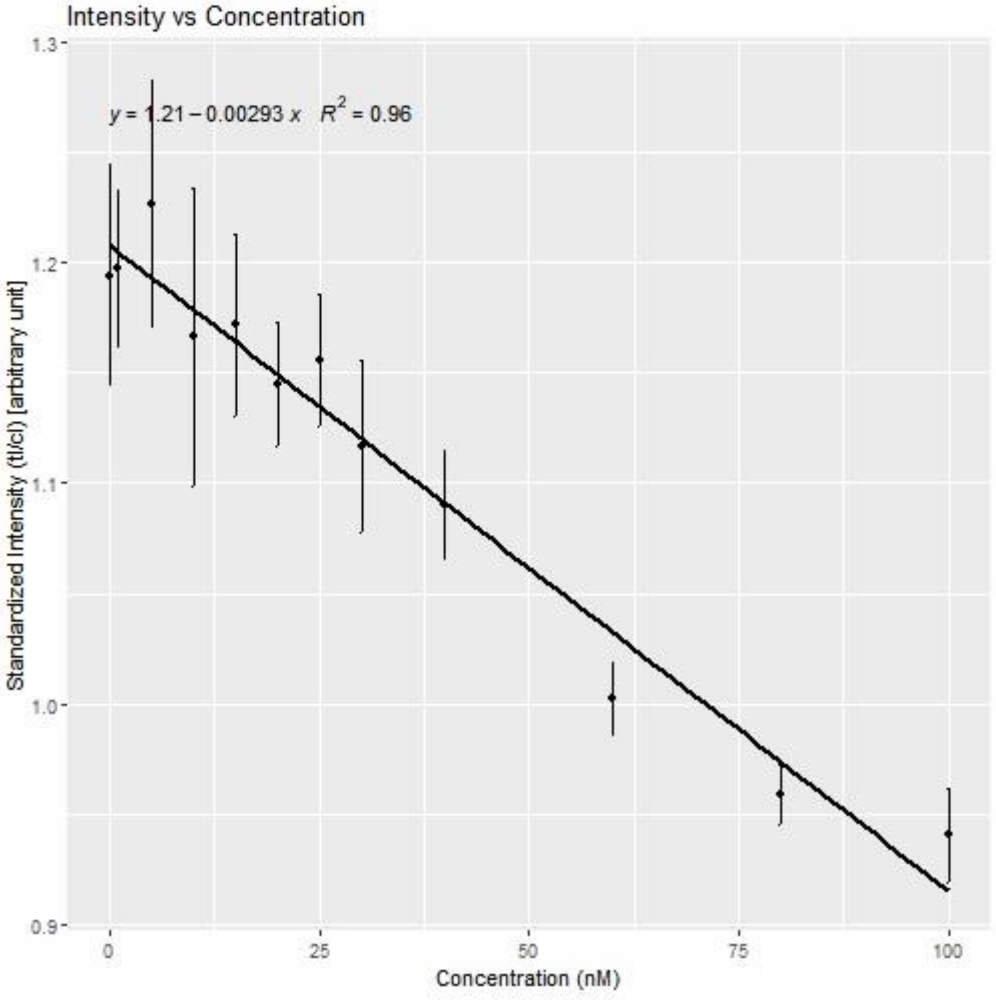

95% Confidence Interval:

**Min.Value**	**Max.Value**
0.8567509	0.953511
1.0582215	1.169336

95% Confidence Interval:

**Min.Value**	**Max.Value**
0.8567509	0.953511
1.0582215	1.169336

Correlation:

**NI_cor**	**SI_cor**
0.9849184	0.9813538

LOD_First Method:

lod_ni	loq_ni	lod_si	loq_si
0.9465362	1.198678	1.003255	1.149937

LOD_Second Method:

lob_ni	lod_ni	loq_ni	lob_si	lod_si	loq_si
0.8977287	0.937441	1.198678	0.9748613	0.9974594	1.149937

Settings used during implementation

Select the type of file: .txt

Intensity value: 1

Slope value: 1

Intercept value: 1

Session Information:

R version 3.5.1 (2018-07-02) Platform: x86_64-w64-mingw32/x64 (64-bit) Running under: Windows >= 8 x64 (build 9200)

Matrix products: default

locale: [1] LC_COLLATE=English_Germany.1252 LC_CTYPE=English_Germany.1252
[3] LC_MONETARY=English_Germany.1252 LC_NUMERIC=C
[5] LC_TIME=English_Germany.1252

attached base packages: character(0)

other attached packages: [1] GNSplex_0.1.0

loaded via a namespace (and not attached): [1] tidyselect_0.2.4 locfit_1.5-9.1 purrr_0.2.5
[4] lattice_0.20-35 colorspace_1.3-2 htmltools_0.3.6
[7] yaml_2.2.0 grDevices_3.5.1 rlang_0.2.2
[10] pillar_1.3.0 later_0.7.5 glue_1.3.0
[13] withr_2.1.2 EBImage_4.22.1 BiocGenerics_0.26.0 [16] RColorBrewer_1.1-2 bindrcpp_0.2.2 jpeg_0.1-8
[19] bindr_0.1.1 plyr_1.8.4 stringr_1.3.1
[22] munsell_0.5.0 gtable_0.2.0 htmlwidgets_1.2
[25] evaluate_0.11 labeling_0.3 Biobase_2.40.0
[28] knitr_1.20 httpuv_1.4.5 parallel_3.5.1
[31] markdown_0.8 highr_0.7 methods_3.5.1
[34] Rcpp_0.12.18 xtable_1.8-3 polynom_1.3-9
[37] ggpmisc_0.3.0 scales_1.0.0 promises_1.0.1
[40] jsonlite_1.5 abind_1.4-5 mime_0.5
[43] ggplot2_3.0.0 stats_3.5.1 datasets_3.5.1

[46] graphics_3.5.1 png_0.1-7 digest_0.6.17
[49] stringi_1.1.7 tiff_0.1-5 dplyr_0.7.6
[52] shiny_1.1.0 grid_3.5.1 tools_3.5.1
[55] bitops_1.0-6 magrittr_1.5 lazyeval_0.2.1
[58] RCurl_1.95-4.11 tibble_1.4.2 crayon_1.3.4
[61] pkgconfig_2.0.2 utils_3.5.1 assertthat_0.2.0
[64] base_3.5.1 rstudioapi_0.7 R6_2.2.2
[67] fftwtools_0.9-8 compiler_3.5.1

S8-Shiny app report: iPhone_GNSplex_Serum

Analysis of the data of lateral flow assay

Initial Data:

Replicate	Test	Control	Conc
R1	1.837941	1.577473	0
R1	1.793863	1.568570	1
R1	1.682933	1.493914	5
R2	1.861876	1.602384	10
R2	1.861303	1.575607	15
R2	1.728782	1.518210	20
R3	1.809022	1.545565	25
R3	1.854788	1.593291	30
R3	1.722505	1.520507	40
R4	1.817732	1.589319	60
R4	1.859975	1.632199	80
R4	1.706194	1.546557	100
R5	1.848091	1.591065	NA
R5	1.827858	1.603789	NA
R5	1.798744	1.587384	NA
R6	1.884875	1.659491	NA
R6	1.765509	1.560062	NA
R6	1.759566	1.589622	NA
R7	1.870799	1.661517	NA
R7	1.870799	1.613687	NA
R7	1.870799	1.553918	NA
R8	1.824395	1.666160	NA
R8	1.846595	1.618664	NA
R8	1.314806	1.229788	NA
R9	1.797113	1.599594	NA
R9	1.602848	1.483151	NA
R9	1.701811	1.597991	NA
R10	1.797271	1.759095	NA
R10	1.735427	1.712039	NA
R10	1.683108	1.644959	NA
R11	1.695619	1.739275	NA
R11	1.728256	1.737603	NA
R11	1.694817	1.690270	NA
R12	1.665508	1.726351	NA
R12	1.746596	1.775527	NA
R12	1.688535	1.727570	NA

Combined replicates:

	NI.crep	SI.crep	NI.sdns	SI.sdns	NI.sd	SI.sd	Concn
R1	0.8734588	1.1450909	0.0147240	0.0193371	0.0798705	0.0458892	0
R2	0.8617775	1.1606544	0.0158756	0.0213425	0.0766770	0.0430052	1
R3	0.8653701	1.1558110	0.0152130	0.0201363	0.0671795	0.0369756	5
R4	0.8861056	1.1288300	0.0176797	0.0222757	0.0794500	0.0428210	10
R5	0.8736112	1.1448018	0.0112778	0.0148652	0.0248063	0.0086079	15
R6	0.8891583	1.1248050	0.0124522	0.0156355	0.0706941	0.0510581	20
R7	0.8604384	1.1630714	0.0288164	0.0391170	0.0000000	0.0539098	25
R8	0.9083907	1.1016388	0.0296875	0.0363034	0.3008248	0.2394093	30
R9	0.9181359	1.0897181	0.0252313	0.0302793	0.0971382	0.0667705	40
R10	0.9808721	1.0195181	0.0049456	0.0051269	0.0571477	0.0573600	60
R11	1.0094906	0.9907369	0.0146477	0.0142964	0.0190787	0.0278229	80
R12	1.0254044	0.9752889	0.0101780	0.0096502	0.0417863	0.0280465	100

Normalized Intensity Plot (Standardized Intensity vs Concentration):

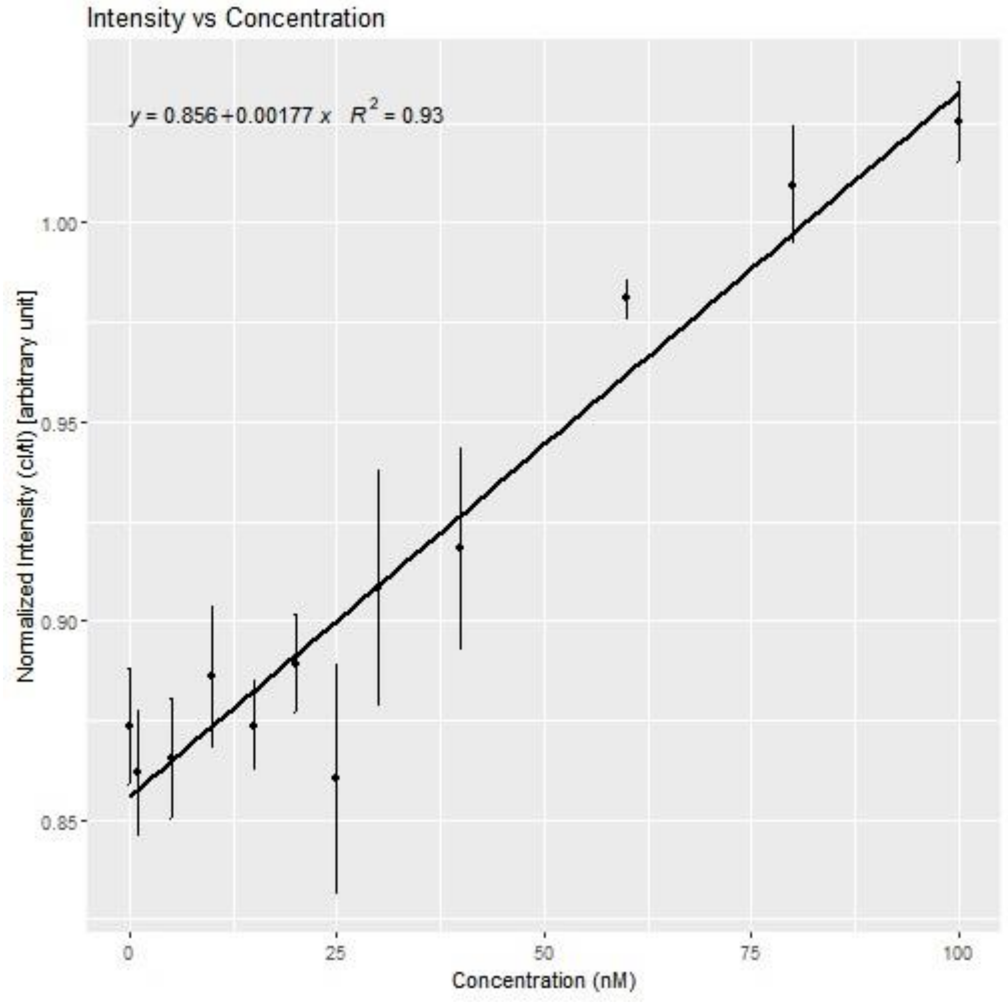

Standardized Intensity Plot (Normalized Intensity vs Concentration):

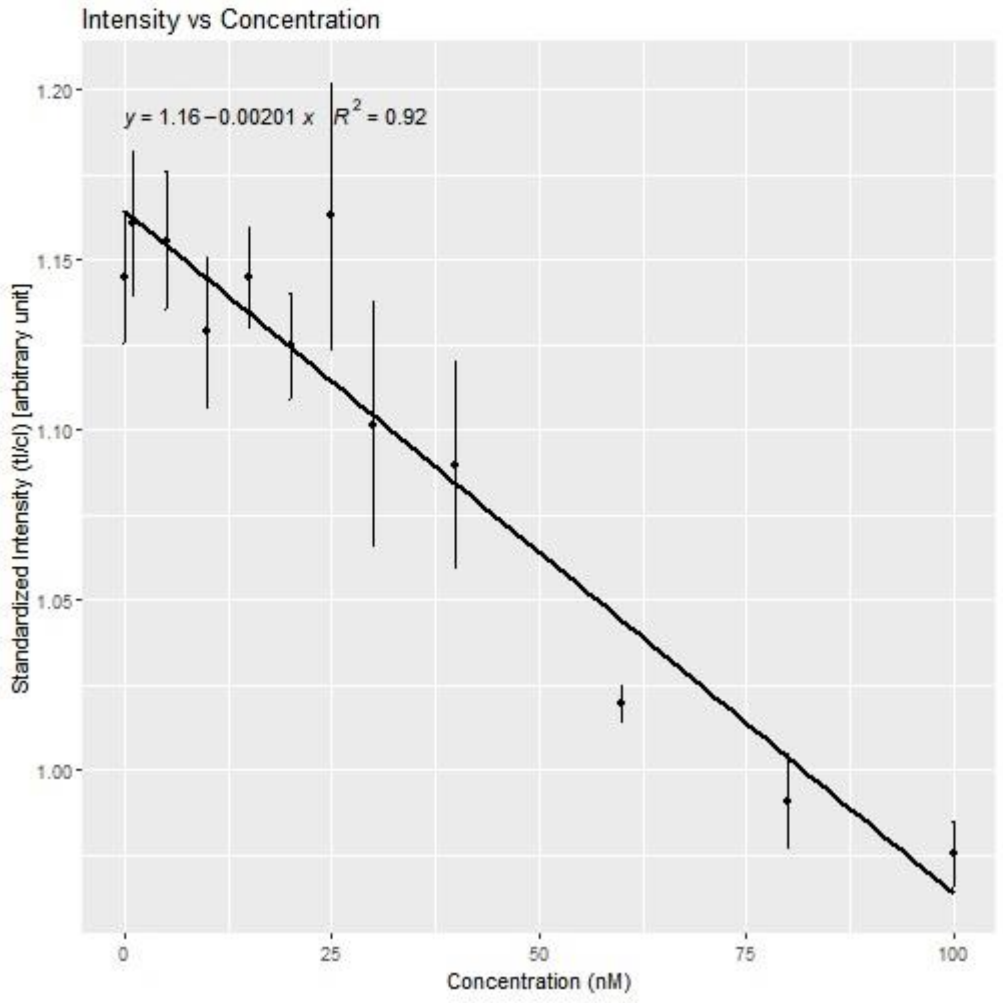

95% Confidence Interval:

Min.Value	Max.Value
0.8784571	0.9469119
1.0609417	1.1390525

95% Confidence Interval:

Min.Value	Max.Value
0.8784571	0.9469119
1.0609417	1.1390525

Correlation:

NI_cor	SI_cor
0.963543	0.9588569

LOD_First Method:

lod_ni	loq_ni	lod_si	loq_si
0.917631	1.020699	1.00424	1.071791

LOD_Second Method:

lob_ni	lod_ni	loq_ni	lob_si	lod_si	loq_si
0.8976799	0.9237953	1.020699	0.9911635	1.014681	1.071791

Settings used during implementation:

Select the type of file: .txt

Intensity value: 1

Slope value: 1

Intercept value: 1

Session Information:

R version 3.5.1 (2018-07-02) Platform: x86_64-w64-mingw32/x64 (64-bit) Running under: Windows >= 8 x64 (build 9200)

Matrix products: default

locale: [1] LC_COLLATE=English_Germany.1252 LC_CTYPE=English_Germany.1252
[3] LC_MONETARY=English_Germany.1252 LC_NUMERIC=C
[5] LC_TIME=English_Germany.1252

attached base packages: character(0)

other attached packages: [1] GNSplex_0.1.0

loaded via a namespace (and not attached): [1] tidyselect_0.2.4 locfit_1.5-9.1 purrr_0.2.5
[4] lattice_0.20-35 colorspace_1.3-2 htmltools_0.3.6
[7] yaml_2.2.0 grDevices_3.5.1 rlang_0.2.2
[10] pillar_1.3.0 later_0.7.5 glue_1.3.0
[13] withr_2.1.2 EBImage_4.22.1 BiocGenerics_0.26.0 [16] RColorBrewer_1.1-2 bindrcpp_0.2.2 jpeg_0.1-8
[19] bindr_0.1.1 plyr_1.8.4 stringr_1.3.1
[22] munsell_0.5.0 gtable_0.2.0 htmlwidgets_1.2
[25] evaluate_0.11 labeling_0.3 Biobase_2.40.0
[28] knitr_1.20 httpuv_1.4.5 parallel_3.5.1
[31] highr_0.7 methods_3.5.1 Rcpp_0.12.18
[34] xtable_1.8-3 polynom_1.3-9 ggpmisc_0.3.0
[37] scales_1.0.0 promises_1.0.1 jsonlite_1.5
[40] abind_1.4-5 mime_0.5 ggplot2_3.0.0
[43] stats_3.5.1 datasets_3.5.1 graphics_3.5.1

[46] png_0.1-7 digest_0.6.17 stringi_1.1.7
[49] tiff_0.1-5 dplyr_0.7.6 shiny_1.1.0
[52] grid_3.5.1 tools_3.5.1 bitops_1.0-6
[55] magrittr_1.5 lazyeval_0.2.1 RCurl_1.95-4.11
[58] tibble_1.4.2 crayon_1.3.4 pkgconfig_2.0.2
[61] utils_3.5.1 assertthat_0.2.0 base_3.5.1
[64] rstudioapi_0.7 R6_2.2.2 fftwtools_0.9-8
[67] compiler_3.5.1
